# Neurotransmitters and neuropeptides in gonadal steroid receptor-expressing cells in medial preoptic area subregions of the male mouse

**DOI:** 10.1038/s41598-017-10213-4

**Published:** 2017-08-29

**Authors:** Yousuke Tsuneoka, Sachine Yoshida, Kenkichi Takase, Satoko Oda, Masaru Kuroda, Hiromasa Funato

**Affiliations:** 1 Department of Anatomy, Faculty of Medicine, Toho University, Tokyo, 143-8540 Japan; 2 PRESTO, Japan Science and Technology Agency, Saitama, 332-0012 Japan; 3 Laboratory of Psychology, Jichi Medical University, Tochigi, 329-0498 Japan; 4 International Institutes for Integrative Sleep Medicine (WPI-IIIS), University of Tsukuba, Ibaraki, 305-8575 Japan

## Abstract

Testosterone is involved in male sexual, parental and aggressive behaviors through the androgen receptor (AR) and estrogen receptor (ER) α expressed in the brain. Although several studies have demonstrated that ERα and AR in the medial preoptic area (MPOA) are required for exhibiting sexual and aggressive behaviors of male mice, the molecular characteristics of ERα- and AR-expressing cells in the mouse MPOA are largely unknown. Here, we performed *in situ* hybridization for neurotransmitters and neuropeptides, combined with immunohistochemistry for ERα and AR to quantitate and characterize gonadal steroid receptor-expressing cells in the MPOA subregions of male mice. *Prodynorphin*, *preproenkephalin* (*Penk*), *cocaine- and amphetamine-related transcript*, *neurotensin*, *galanin*, *tachykinin* (*Tac*)1, *Tac2* and *thyrotropin releasing hormone* (*Trh*) have distinct expression patterns in the MPOA subregions. *Gad67*-expressing cells were the most dominant neuronal subtype among the ERα- and AR-expressing cells throughout the MPOA. The percentage of ERα- and AR-immunoreactivities varied depending on the neuronal subtype. A substantial proportion of the *neurotensin*-, *galanin*-, *Tac2*- and *Penk*-expressing cells in the MPOA were positive for ERα and AR, whereas the vast majority of the *Trh*-expressing cells were negative. These results suggest that testosterone exerts differential effects depending on both the neuronal subtypes and MPOA subregions.

## Introduction

Androgens such as testosterone play a central role in the regulation of the sexual, parental and aggressive behaviors of male animals through a direct action on androgen receptors (AR) and an indirect action on estrogen receptors (ERs), such as ERα and ERβ after being aromatized into estradiol in the brain^1–4^. The medial preoptic area (MPOA), the most anterior part of the hypothalamus, is one of the brain regions with the most abundant expression of AR and ERα^5–8^ and regulates the sexual, parental and aggressive behaviors of rodents and humans^2, 9–14^. Importantly, the MPOA abundantly expresses aromatase, which converts testosterone into estradiol^15^. The local injection of an aromatase inhibitor into the MPOA suppressed male sexual behaviors^16^.

The MPOA is not a homogeneous structure, and it exhibits regional differences in terms of the neuron density, the distribution of neuron subtypes characterized by gene expression, and the spreading of afferent fibers, such as 5-HT-immunoreactive fibers^10, 17–20^. We recently substantiated the functional relevance of the MPOA subregions by showing subregion-specific neuronal activation in response to aggression, ejaculation, paternal behavior and infanticide^10^, as well as in response to maternal behaviors^18^.

In addition to ERα and AR, various neuropeptides, such as cocaine- and amphetamine-related transcript (Cart), dynorphin, enkephalin, galanin, neurotensin, substance P (encoded by *Tac1*), neurokinin B (encoded by *Tac2*) and thyrotropin releasing hormone (Trh) are expressed in the MPOA^17, 18, 21–23^. A recent report showed that the ablation of galanin-expressing neurons in the MPOA inhibited paternal behaviors, and the optogenetic activation of galanin-expressing neurons enhanced paternal behaviors towards pups^24^, which suggests that neuronal subtypes characterized by the differential expressions of neuropeptides and neurotransmitters in the MPOA may have a distinct role in sexual and parental behaviors.

To further understand how specific neuronal groups are involved in gonadal steroid-dependent reproductive behaviors, it is crucial to identify the expression of the neurotransmitters and neuropeptides of ERα- and AR-expressing cells in each MPOA subregion. In this study, we quantitated the expression of neurotransmitters and neuropeptides for ERα- or AR-expressing cells in each MPOA subregion of male mice, and the expression was delineated using MPOA subregion markers, such as calbindin, oxytocin *neurotensin*, *preproenkephalin* (*Penk*) and *vesicular glutamate transporter2* (*Vglut2*)^17, 18, 25, 26^.

## Results

### MPOA subregions

**Figure 1:**
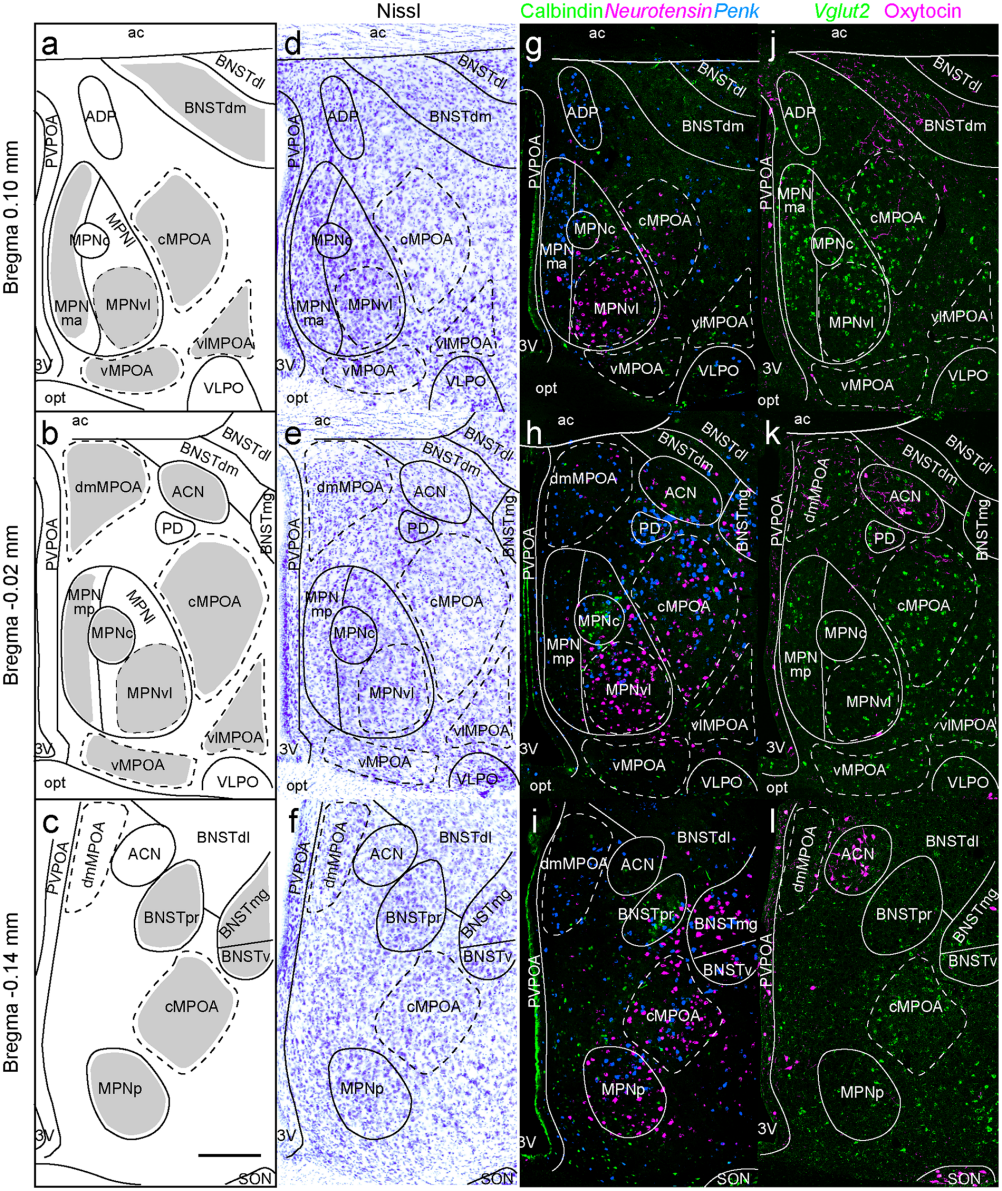
Medial preoptic area (MPOA) subregions identified on Nissl-stained sections and fluorescent images using area markers. (**a–c)** Schematic drawings showing the MPOA subregions and nuclei of the bed nucleus of the stria terminalis (BNST). The shaded areas in (**a–c**) were quantitated for gonadal hormone receptor-expressing cells in this study. (**d–f**) Representative images of Nissl-stained coronal sections along the anterior-posterior axis. (**g–i**) Representative images of calbindin (green), *Neurotensin* (magenta) and *Penk* (light blue). (**j–l**) Representative images of *Vglut2* (green) and oxytocin (magenta). These images are representative of at least six different mice for each image series. Scale bars: 200 μm. 3 v, third ventricle; ac, anterior commissure; ACN, anterior commissural nucleus; ADP, anterodorsal preoptic nucleus; BNSTdm, dorsomedial nucleus of the BNST; BNSTmg, magnocellular nucleus of the BNST; BNSTpr, principal nucleus of the BNST; BNSTv, ventral nucleus of the BNST; cMPOA, central part of the MPOA; dmMPOA, dorsomedial part of the MPOA; MPNc, central part of the MPN; MPNma, anteromedial part of the MPN; MPNmp, posteromedial part of the MPN; MPNp, posterior part of the MPN; MPNvl, ventrolateral part of the MPN; opt, optic tract; PD, posterodorsal preoptic nucleus; SON, supraoptic nucleus; vMPOA, ventral part of the MPOA; vlMPOA, ventrolateral part of the MPOA; VLPO, ventrolateral preoptic nucleus.

The MPOA subregions were identified as previously described^10, 17–19, 22, 26–28^. Although the brain atlas identified several nuclei in the POA region, such as the medial preoptic nucleus (MPN), posterodorsal preoptic nucleus (PD) and ventrolateral preoptic nucleus (VLPO), the relatively large region outside of these nuclei sandwiched between the anterior commissure and the optic tract remains unnamed or is collectively referred to as the MPOA (Allen brain atlas: http://www.brain-map.org/)^29^. Thus, in our study, we subdivided this broad MPOA into four regions: the dorsomedial part of the MPOA (dmMPOA), central part of the MPOA (cMPOA), ventral part of the MPOA (vMPOA), and ventrolateral part of the MPOA (vlMPOA). These MPOA subregions were easily identified on Nissl-stained sections and fluorescent images according to the location (Fig. 1).

In general, the subregions and nuclei that were identified on Nissl-stained sections were also easily identified on fluorescent images based on their locations and gene expressions. The central part of the MPN (MPNc) has been shown to predominantly overlapped with the sexually dimorphic nucleus of the preoptic area in rats^30, 31^, which can be identified by a dense cluster of calbindin-positive cells in rats and mice^6, 26, 32^ (Fig. 1h). The lateral subdivision of the MPN (MPNl) has a cluster of *neurotensin*-positive cells^17, 18^. Since *neurotensin* was intensely expressed in the ventral part of the MPNl (MPNvl), we assessed for AR- and ERα-positive cells in the MPNvl (Fig. 1g,h). The anterior commissural nucleus (ACN) was characterized by a population of oxytocinergic neurons in the dorsal MPOA^25, 29^.

In some cases, gene expressions enable us to subdivide Nissl-based MPN structures. The MPNm was further divided into the anterior part (MPNma), which contains a cluster of *Penk*-expressing cells, and the posterior part (MPNmp), which has a higher density of *Cart*-expressing cells, arbitrarily at 0.04 mm anterior to the bregma (Figs 1 and 3). The most posterior part of the MPNl (MPNp) was different from the main part of the MPNl in the densities of *neurotensin-*, *Penk-*, *prodynorphin* (*Pdyn*)*-* and *Tac1-*positive cells (Figs 1, 3 and 4).

In addition to the MPOA subregions, we quantitated the AR- and ERα-positive cells in the dorsomedial nucleus of the BNST (BNSTdm), principal nucleus of the BNST (BNSTpr), ventral nucleus of the BNST (BNSTv) and magnocellular nucleus of the BNST (BNSTmg), which are located near the MPOA. We identified BNST nuclei as previously described^19, 33, 34^ and followed their nomenclature. Similar to the MPOA, the BNST nuclei identified on Nissl-stained sections were easily identified on fluorescent images based on their locations and gene expressions. The BNSTpr was identified by the presence of abundant calbindin-positive neurons in its core area^35, 36^. The BNSTdm was located ventrally to the anterior commissure and was characterized by moderate oxytocin-ir fibers^17^. Since the BNSTv and BNSTmg were commonly identified with moderately dense *neurotensin*-positive cells^19^, we quantitated for BNSTv and BNSTmg together, subsequently referred to as BNSTv/mg.

### Regional differences in ERα-ir and AR-ir cell densities

**Figure 2:**
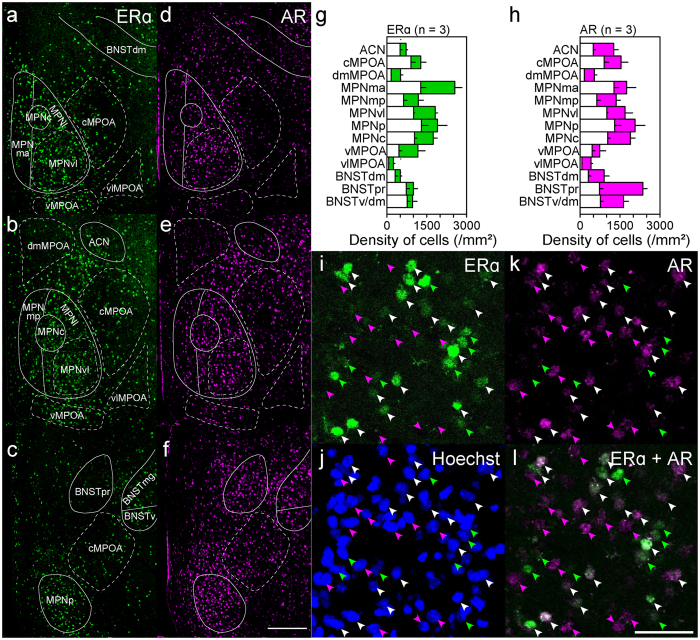
Distributions of ERα- and AR-ir cells in the MPOA and adjacent areas in male mice. (**a–c**) Representative images of single immunostaining for ERα of the MPOA. (**d–f**) Representative images of single immunostaining for AR of the MPOA. Solid and dashed lines indicate delineations of the quantified subregions. (**a**,**d**) Bregma, +0.10 mm. (**b**,**e**) Bregma, −0.02 mm. (**c**,**f**) Bregma, −0.14 mm. (**g**,**h**) Regional differences in singly or doubly positive cells examined with double immunostaining for ERα and AR. Open bars indicate the density of cells doubly positive for ERα and AR. Filled bars indicate the density of cells singly positive for ERα (**g**) or AR (**h**) Three sections derived from different mice were counted. (**i–l**) Representative fluorescent images of double fluorescent immunohistochemistry for ERα and AR. (**i**) ERα (**j**) AR. (**k**) Hoechst. (**l**) Merge of ERα and AR. Scale bars: 200 μm in (**f**), 50 μm in, (**o**).

First, we performed immunostaining for ERα or AR to examine the cell density of gonadal steroid receptor-positive cells in each MPOA subregion. ERα-ir cells were densest in the MPNma, followed by the MPNvl, MPNp and MPNc. ERα-ir cells were sparse in the dmMPOA, vlMPOA and BNSTdm (n = 3, Fig. 2a–c,g). Dense AR-ir cells were found in the ACN, cMPOA, MPNvl, MPNp, MPNma, MPNmp, MPNc, BNSTpr and BNSTv/mg. Similar to ERα-ir cells, AR-ir cells were sparse in the dmMPOA and vlMPOA (n = 3, Fig. 2d–f,h). The largest difference in the cell densities of ERα-ir cells and AR-ir cells was recognized in the BNSTpr, which contains a larger density of AR-ir cells than ERα-ir cells.

To examine the coexpression of ERα and AR, we performed double immunostaining for both receptors. Cells that expressed both ERα and AR were observed throughout the MPOA (n = 3, Fig. 2g,h, Supplementary information Table S2). Double-labeled cells were dense in the MPNma, MPNp, MPNvl and MPNc. In general, approximately half of the ERα-ir cells in the MPOA were immunoreactive for AR. ERα-ir cells in the ACN, cMPOA, MPNp, BNSTpr and BNSTv/mg showed a high proportion of double-labeling (68.0–79.5%). In the MPNma, MPNvl and MPNp, a substantial proportion of AR-ir cells was also immunoreactive for ERα (61.7–74.0%).

### Neurotransmitters and neuropeptides in MPOA subregions

**Figure 3:**
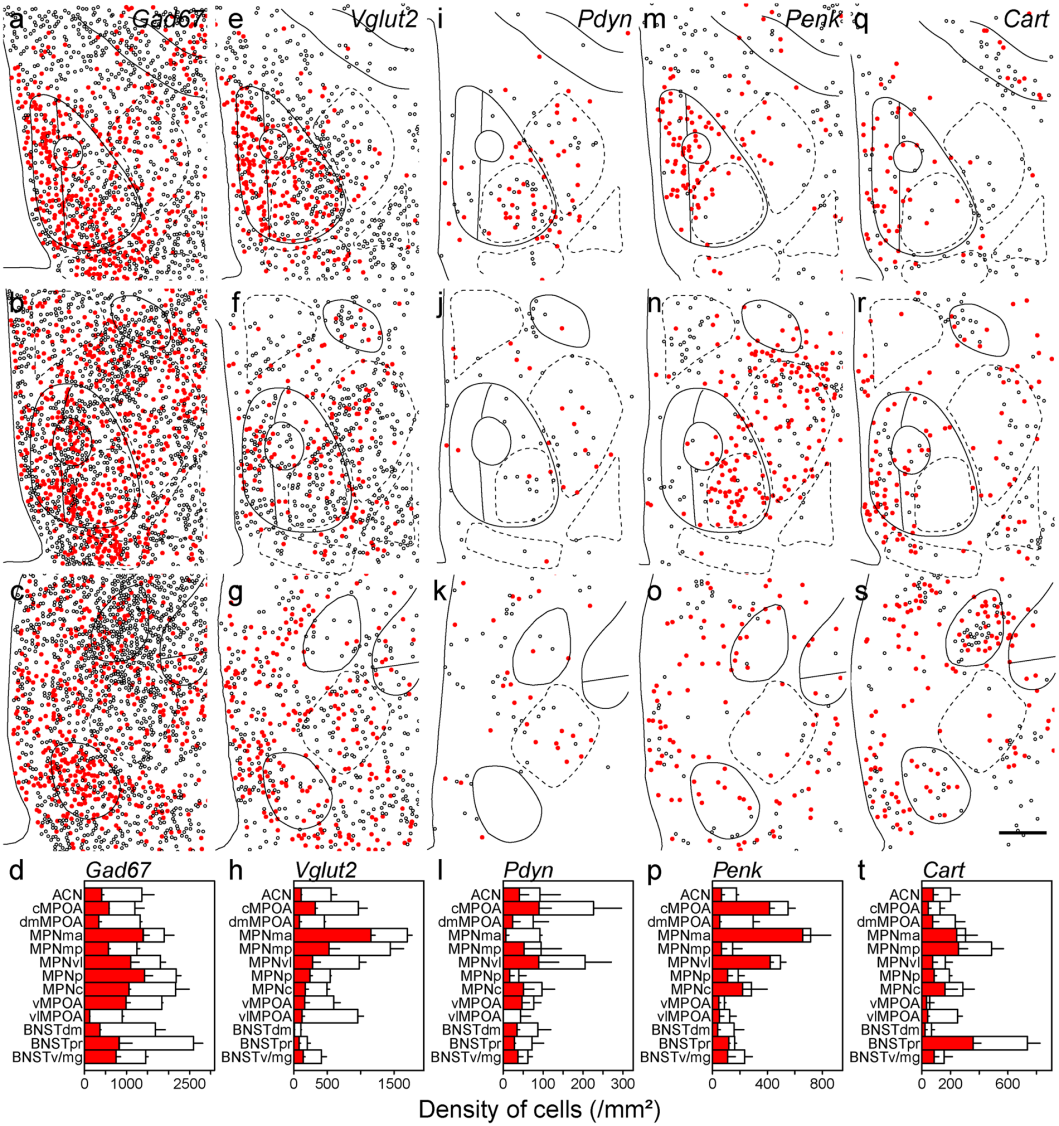
Distributions of singly and doubly positive cells of *in situ* hybridization for neurotransmitters and neuropeptides with immunostaining for ERα in the MPOA and adjacent areas.Representative cell distributions of *in situ* hybridization for *Gad67* (**a–c**, n = 4), *Vglut2* (**e–g**, n = 3), *Pdyn* (**i–k**, n = 3), *Penk* (**m–o**, n = 3) and *Cart* (**q–s**, n = 3), combined with immunostaining for ERα. Filled circles indicate cells doubly positive for neurotransmitter/neuropeptide and ERα. Open circles indicate neurotransmitter/neuropeptide-positive and ERα-negative cells. Solid and dashed lines indicate delineations of the quantified subregions. The third ventricle is located on the left side of each panel. (**d**,**h**,**l**,**p**,**t**) Filled bars indicate cells doubly positive for neurotransmitter/neuropeptide and ERα (mean ± S. E.). Open bars indicate cells positive for neurotransmitter/neuropeptide-positive and negative for ERα (mean ± S. E.). (**d**) *Gad67*. (**h**) *Vglut2*. (**l**) *Pdyn*. (**p**) *Penk*. (**t**) *Cart*. (**a**,**e**,**i**,**m**,**q**) Bregma, + 0.10 mm. (**b**,**f**,**j**,**n**,**r**) Bregma, −0.02 mm mm. (**c**,**g**,**k**,**o**,**s**) Bregma, −0.14 mm. Scale bars: 200 μm.

**Figure 4:**
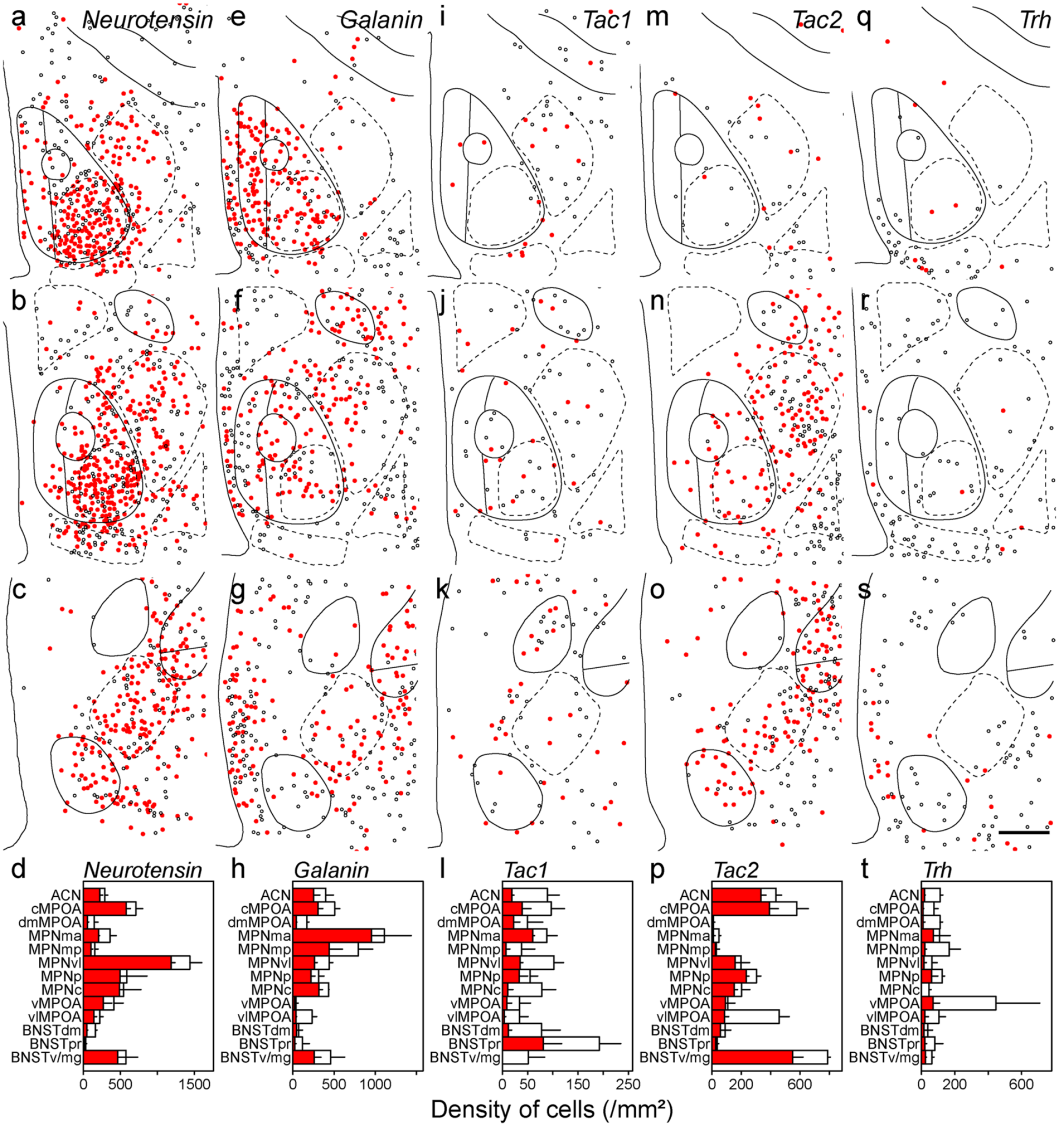
Distributions of singly and doubly positive cells of *in situ* hybridization for neurotransmitters and neuropeptides with immunostaining for ERα in the MPOA and adjacent areas. Representative cell distributions of *in situ* hybridization for Neurotensin (**a**–**c**, n = 3), Galanin (**e**–**g**, n = 3), Tac1 (**i**–**k**, n = 3), Tac2 (**m**–**o**, n = 3) and Trh (**q**–**s**, n = 3), combined with immunostaining for ERα. Filled circles indicate cells doubly positive for neurotransmitter/neuropeptide and ERα. Open circles indicate neurotransmitter/neuropeptide-positive and ERα-negative cells. Solid and dashed lines indicate delineations of the quantified subregions. The third ventricle is located on the left side of each panel. (**d**,**h**,**l**,**p**,**t**) Filled bars indicate cells doubly positive for neurotransmitter/neuropeptide and ERα (mean ± S. E.). Open bars indicate cells positive for neurotransmitter/neuropeptide-positive and negative for ERα (mean ± S. E.). (**d**) *Neurotensin*. (**h**) *Galanin*. (**l**) *Tac1*. (**p**) *Tac2*. (**t**) *Trh*. (**a**,**e**,**i**,**m**,**q**) Bregma, + 0.10 mm. (**b**,**f**,**j**,**n**,**r**): Bregma, −0.02 mm. (**c**,**g**,**k**,**o**,**s**) Bregma, −0.14 mm. Scale bars: 200 μm.

Since the MPOA is rich in GABAergic neurons, *glutamate decarboxylase 67* (*Gad67*)-expressing cells were found in the entire MPOA with a high density in the BNSTpr, MPNp and MPNc and a low density in the vlMPOA (Fig. 3a–c). In general, the cell density of *Vglut2*-expressing cells was lower than that of *Gad67*-expressing cells in the MPOA (Fig. 3a–h). The *Vglut2*-expressing cell density was higher in the cMPOA and ventromedial region of the MPOA including the MPNma and MPNmp, and was lower in the dorsal MPOA such as the ACN and dmMPOA, and the BNSTdm, BNSTpr and BNSTv/mg (Fig. 3e–h). A low density of *Pdyn*-expressing cells was mainly found in the cMPOA and MPNvl (Fig. 3i–l). *Penk*-positive cells were abundant in the MPNma, cMPOA, dmMPOA and MPNvl (Fig. 3m–p). The highest density of *Penk*-positive cells was found at the border area between the cMPOA and ACN (Fig. 1). *Cart*-expressing cell clusters were recognized in the BNSTpr and MPNmp (Fig. 3q–t). The vMPOA and dmBNST contained very few *Cart*-expressing cells. *Neurotensin*-expressing cells were abundant in the MPNvl and cMPOA, and they were sparse in the BNSTpr, dmMPOA and MPNmp (Fig. 4a–d). The distribution of *neurotensin*-expressing cells showed a ventrolateral to dorsomedial gradient. The density of *galanin*-expressing cells was higher in the MPNma, MPNmp and PVPOA and lower in the dmMPOA, vMPOA, BNSTdm and BNSTpr (Fig. 4e–h). A low density of *Tac1*-expressing cells was scattered throughout the entire MPOA with the highest density in the BNSTpr (Fig. 4i–l). The distribution of *Tac2*-expressing cells showed a posterolateral-anteromedial gradient. The BNSTv/mg, cMPOA, ACN and vlMPOA had abundant *Tac2*-expressing cells whereas very few *Tac2*-expressing cells were found in the dmMPOA, MPNma, MPNmp or BNSTpr (Fig. 4m–p). *Trh*-expressing cells were distributed mainly in the periphery of the MPOA with the highest cell density in the vMPOA (Fig. 4q–t).

### Neurotransmitters and neuropeptides of ERα-positive cells

**Figure 5:**
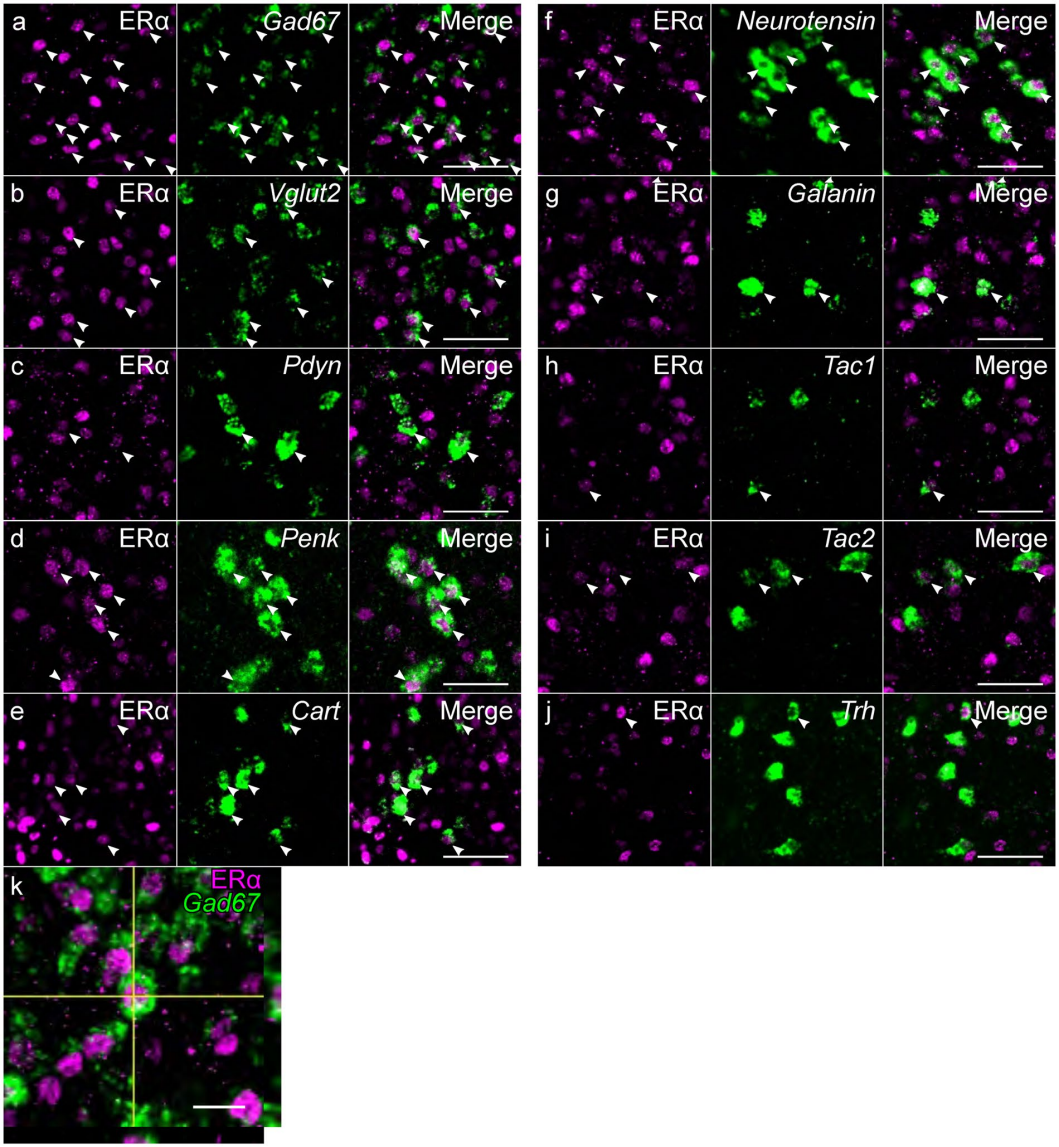
Representative fluorescent images of *in situ* hybridization for neurotransmitters and neuropeptides combined with immunostaining for ERα (**a**). *Gad67*. (**b**) *Vglut2*. (**c**) *Pdyn*. (**d**) *Penk*. (**e**) *Cart*. (**f**) *Neurotensin*. (**g**) *Galanin*. (**h**) *Tac1*. (**i**) *Tac2* (**j**) *Trh*. (**k**) Orthogonal view of *Gad67* and ERα positive cells. Each image series is representative of at least three different mice. Arrowheads indicate double positive cells. Scale bars: 50 μm (**a**–**j**), 20 μm (**k**).

In general, approximately half of the *Gad67*-expressing cells of the MPOA were immunoreactive for ERα. The percentage of ERα-positive cells among *Gad67*-expressing cells was higher in the MPNma and MPNp, and lower in the vlMPOA, ACN, dmMPOA and BNSTdm (n = 4, Figs 3a–d and 5a,k). The percentage of ERα-positive cells among *Vglut2*-expressing cells was also highest in the MPNma and lowest in the ACN, dmMPOA and vlMPOA (n = 3, Figs 3e–h, 5b). The density of cells that expressed *Pdyn* and ERα was higher in the cMPOA and MPNvl (n = 3, Figs 3i–l, 5c). Cells that doubly expressed *Penk* and ERα were abundant in the MPNma, cMPOA and MPNvl (n = 3, Figs 3m–p, 5d). Of note, a vast majority of *Penk*-expressing cells were positive for ERα in the MPNma. The percentage of ERα-immunoreactive cells among *Cart*-positive cells was higher in the MPNma and lower in the vlMPOA (n = 3, Figs 3q–t, 5e). Overall, more than 80% of *neurotensin*-positive cells were immunoreactive for ERα in the entire MPOA (n = 3, Figs 4a–d, 5f). The MPNma contained the highest density of cells that expressed both ERα and *galanin* (n = 3, Figs 4e–h, 5g). Overall, less than half of *Tac1*-expressing cells were ERα-immunoreactive in the MPOA (n = 3, Figs 4i–l, 5h). Seventy to ninety percent of *Tac2*-expressing cells were positive for ERα in the MPOA except for the vlMPOA (n = 3, Figs 4m–p, 5i). Most *Trh*-expressing cells did not express ERα (n = 3, Figs 4q–t, 5j).

### Neurotransmitters and neuropeptides of ERα cells in MPOA subregions

Next, we quantitated the proportion of neurotransmitter/neuropeptide- and ERα- doubly positive cells in each neurotransmitter/neuropeptide-positive cell and the proportion of the doubly positive cells in ERα-immunoreactive cells for each MPOA subregion (Figs 3–4, Supplementary information Table S3). In the ACN, more than half of the ERα-immunoreactive cells were *Gad67*, *galanin* and *Tac2* positive cells. In the cMPOA, a large proportion of the ERα-immunoreactive cells expressed *Gad67*, *neurotensin* and *galanin*. In the MPNma, the large proportion of the ERα-immunoreactive cells expressed *Gad67* and *Vglut2*. MPNmp was characterized by the large proportion of *Vglut2*-expression in ERα-immunoreactive cells. In the MPNvl, the majority of the ERα-positive cells were *Gad67*- and *neurotensin*-expressing cells. In the MPNp, MPNc, vMPOA BNSTdm, BNSTpr and BNSTv/mg, most ERα-immunoreactive cells were *Gad67*-expressing cells. The vlMPOA showed the least proportion of *Gad67* expression in ERα-immunoreactive cells among the MPOA subregions. In the MPNp, SDN-POA, vMPOA BNSTdm, BNSTpr and BNSTv/mg, most ERα-immunoreactive cells were *Gad67*-expressing cells. In the BNSTv/mg, the majority of the ERα-immunoreactive cells were *Gad67*-expressing cells, followed by *galanin*- and *Tac2*-expressing cells.

### Neurotransmitters and neuropeptides of AR-positive cells

**Figure 6:**
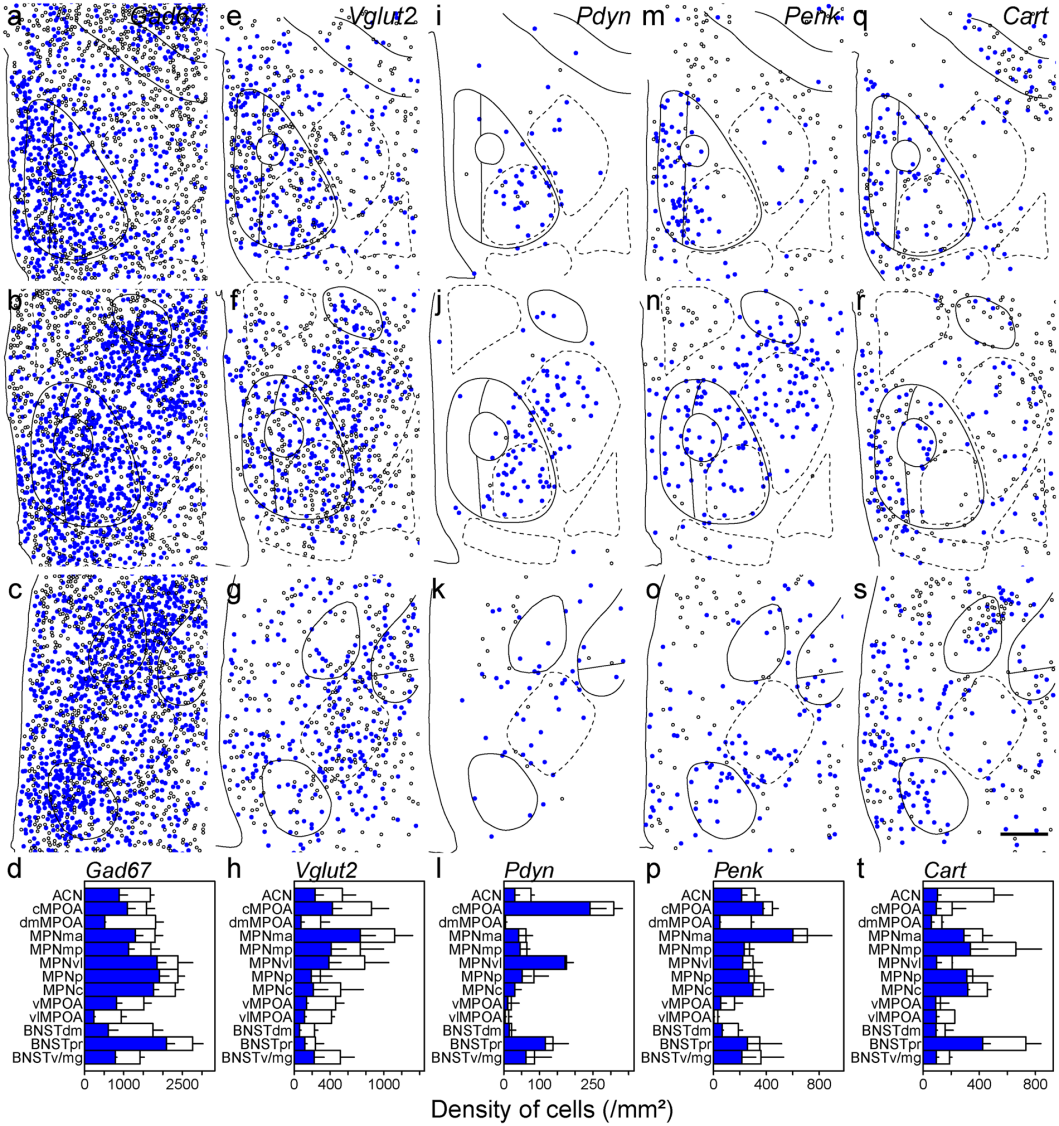
Distributions of singly and doubly positive cells of *in situ* hybridization for neurotransmitters and neuropeptides with immunostaining for AR in the MPOA and adjacent areas.Representative cell distributions of *in situ* hybridization for *Gad67* (**a–c**, n = 3), *Vglut2* (**e–g**, n = 3), *Pdyn* (**i**–**k**, n = 3), *Penk* (**m**–**o**, n = 3) and *Cart* (**q**–**s**, n = 4), combined with immunostaining for AR. Filled circles indicate cells doubly positive for neurotransmitter/neuropeptide and AR. Open circles indicate neurotransmitter/neuropeptide-positive and AR-negative cells. Solid and dashed lines indicate delineations of the quantified subregions. The third ventricle is located on the left side of each panel. (**d**,**h**,**l**,**p**,**t**) Filled bars indicate cells doubly positive for neurotransmitter/neuropeptide and AR (mean ± S. E.). Open bars indicate cells positive for neurotransmitter/neuropeptide-positive and negative for AR (mean ± S. E.). (**d**) *Gad67*. (**h**) *Vglut2*. (**l**) *Pdyn*. (**p**) *Penk*. (**t**) *Cart*. (**a**,**e**,**i**,**m**,**q**) Bregma, + 0.10 mm. (**b**,**f**,**j**,**n**,**r**) Bregma −0.02 mm. (**c**,**g**,**k**,**o**,**s**): Bregma, −0.14 mm. Scale bars: 200 μm.

**Figure 7:**
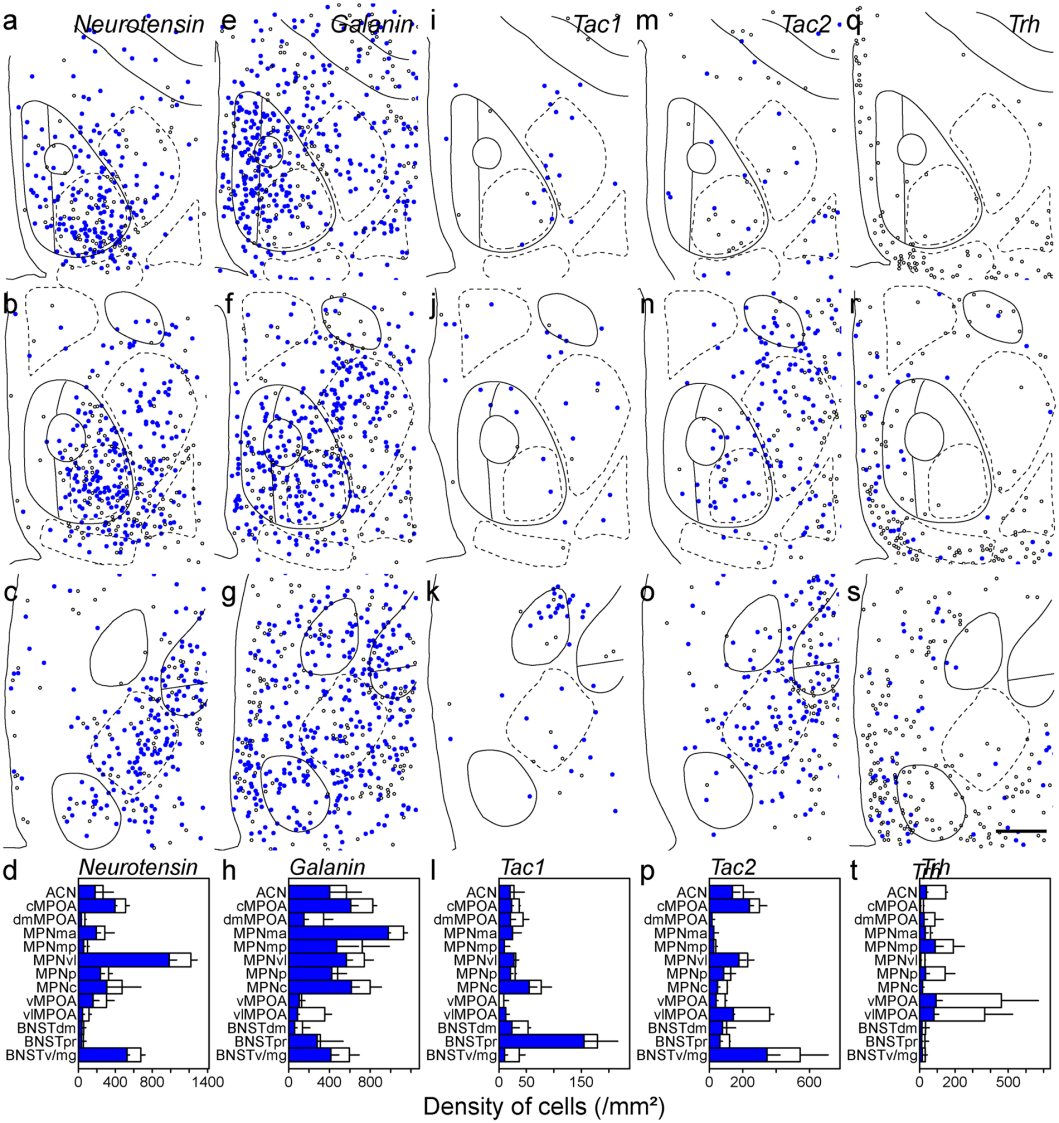
Distributions of singly and doubly positive cells of *in situ* hybridization for neurotransmitters and neuropeptides with immunostaining for AR in the MPOA and adjacent areas.Representative cell distributions of *in situ* hybridization for *Neurotensin* (**a**–**c**, n = 3), *Galanin* (**e**–**g**, n = 3), *Tac1* (**i**–**k**, n = 3), *Tac2* (**m**–**o**, n = 4), and *Trh* (**q**–**s**, n = 3), combined with immunostaining for AR. Filled circles indicate cells doubly positive for neurotransmitter/neuropeptide and AR. Open circles indicate neurotransmitter/neuropeptide-positive and AR-negative cells. Solid and dashed lines indicate delineations of the quantified subregions. The third ventricle is located on the left side of each panel. (**d**) *Neurotensin*. (**h**) *Galanin*. (**l**) *Tac1*. (**p**) *Tac2*. (**t**) *Trh*. (**a**,**e**,**i**,**m**,**q**): Bregma, + 0.10 mm. (**b**,**f**,**j**,**n**,**r**) Bregma, −0.02 mm. (**c**,**g**,**k**,**o**,**s**) Bregma, −0.14 mm. Scale bars: 200 μm.

**Figure 8:**
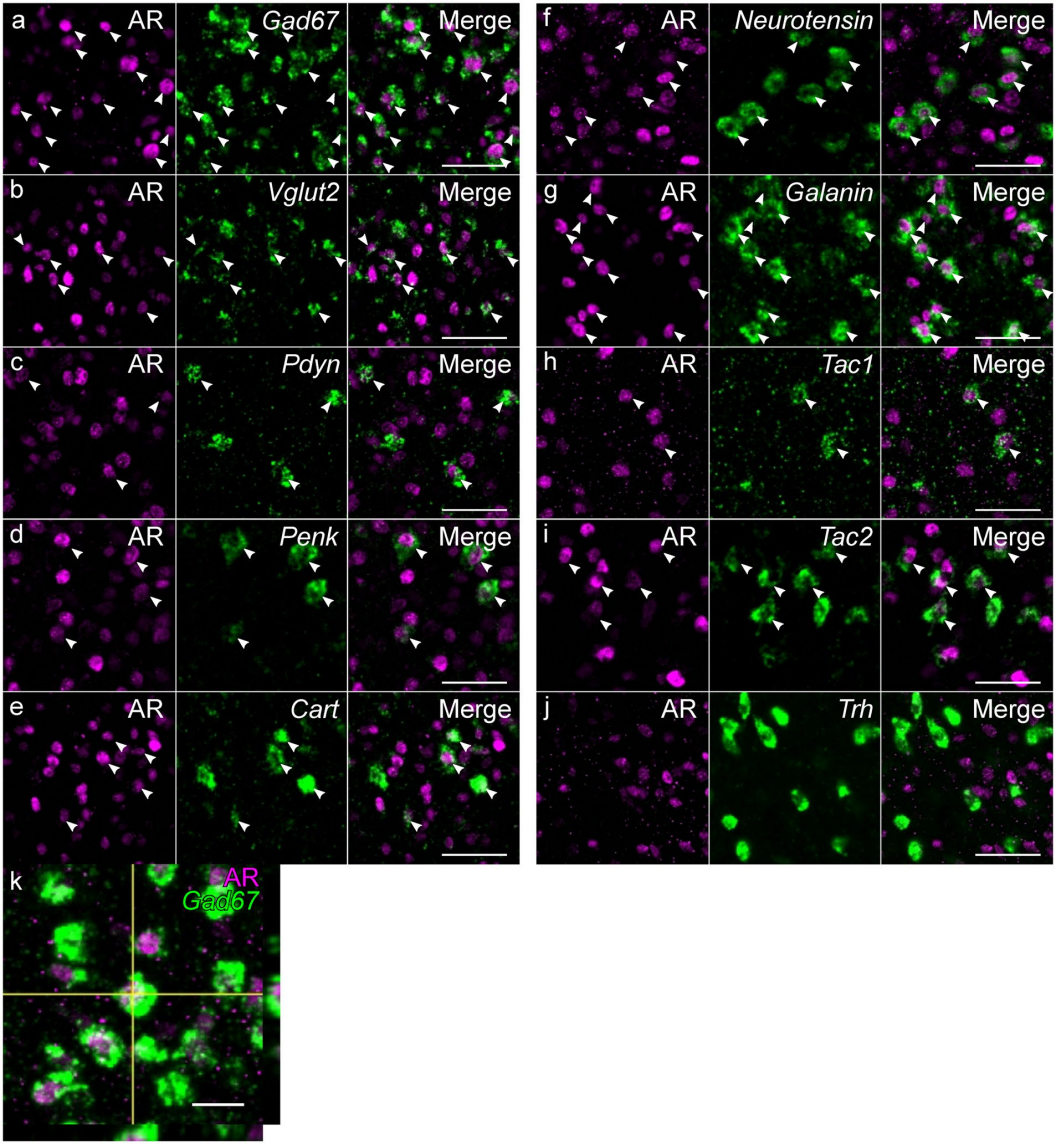
Representative fluorescent images of *in situ* hybridization for neurotransmitters and neuropeptides combined with immunostaining for AR. (**a**) *Gad67*. (**b**) *Vglut2*. (**c**) *Pdyn*. (**d**) *Penk*. (**e**) *Cart*. (**f**) *Neurotensin*. (**g**) *Galanin*. (**h**) *Tac1*. (**i**)*Tac2*. (**j**) *Trh*. (**k**) Orthogonal view of *Gad67* and AR positive cells. Each image series is representative of at least three different mice. Arrowheads indicate double positive cells. Scale bars: 50 μm (**a**–**j**), 20 μm (**k**).

The proportion of AR-positive cells in *Gad67*-expressing cells was higher in the MPNvl, MPNp and MPNc, and lower in the dmMPOA, vlMPOA and BNSTdm (n = 3, Figs 6a–d, 8a,k). The proportion of AR-positive cells in *Vglut2*-expressing cells was highest in the MPNma and lower in the vlMPOA, dmMPOA and BNSTdm (n = 3, Figs 6e–h, 8b). In contrast to low ERα-immunoreactivity (Fig. 4i–l), a high proportion of *Pdyn*-expressing cells were AR-positive cells in the entire MPOA (n = 3, Figs 6i–l, 8c). The proportion of AR-immunoreactive cells in *Penk*-expressing cells was higher in the cMPOA, MPNma, MPNmp and MPNp, and lower in the dmMPOA (n = 3, Figs 6m–p, 8d). The proportion of AR-immunoreactive cells in *Cart*-expressing cells was highest in the MPNp and lowest in the ACN (n = 4, Figs 6q–t, 8e). Similar to ERα (Fig. 4a–d), approximately 80% of *neurotensin*-expressing cells were immunoreactive for AR in the entire MPOA (n = 3, Figs 7a–d, 8f). Most *galanin*-expressing cells were immunoreactive for AR in the ACN, cMPOA, MPNma, MPNvl, MPNp, MPNc, vMPOA, BNSTpr and BNSTv/mg (n = 3, Figs 7e–h, 8g). Cells that expressed both AR and *Tac1* were abundantly found in the BNSTpr (n = 3, Figs 7i–l, 8h). Similar to ERα (Fig. 4m–p), the proportion of AR-immunoreactive cells in *Tac2*-expressing cells was higher in the cMPOA and MPNvl, and was lower in the vlMPOA (n = 4, Figs 7m–p, 7i). Among the neurotransmitters and neuropeptides examined, *Trh*-expressing cells were the lowest proportion of AR-immunoreactive cells in all MPOA subregions (n = 3, Figs 7q–t, 8j).

### Neurotransmitters and neuropeptides of AR cells in MPOA subregions

Lastly, we quantitated the proportion of neurotransmitter/neuropeptide- and AR- doubly positive cells in each neurotransmitter/neuropeptide-positive cell and the proportion of the doubly positive cells in AR-immunoreactive cells for each MPOA subregion (Figs 6–7, Supplementary information Table S4). In the cMPOA, a large proportion of AR-positive cells expressed *Gad67*, *galanin* and *neurotensin*. In the MPNma, the majority of AR-positive cells expressed *Gad67* and *galanin*. In the MPNmp, half of the AR-positive cells were *Gad67*-expressing cells, followed by *galanin*- and *Cart*-expressing cells. In the MPNvl, the majority of the AR-positive cells were *Gad67*- and *neurotensin*-expressing cells. Since AR-ir cells were sparse in the vlMPOA, the cell density of double-labeled AR-immunoreactive cells was low for all neurotransmitters and neuropeptides examined. In the BNSTv/mg, the majority of ARα-immunoreactive cells expressed *Gad67* and *neurotensin*.

## Discussion

The present results show that gonadal steroid receptor-positive cells are not homogeneously distributed throughout the MPOA of male mice. The density of cells that express different neuropeptides and neurotransmitters largely differs among the MPOA subregions. Moreover, the proportions of cells that expressed ERα and AR were different among each neurotransmitter/neuropeptide. In addition to our previous study using female mice^10, 18^, these results showed the subregion heterogeneity of the MPOA. The current morphological data imply that *neurotensin*-, *galanin*-, *Tac2*- and *Penk-*expressing cells may be sensitive to gonadal steroid hormones whereas *Trh-*expressing cells are the least sensitive to AR and ERα.

The quantitation of ERα- and AR-positive cells clearly showed a differential density of gonadal steroid receptor-expressing cells in each MPOA subregion. The highest densities of ERα- and AR-positive cells were found in the MPNma and BNSTpr, respectively. In contrast, the dmMPOA, vlMPOA and BNSTdm contained the lower densities of ERα- and AR-positive cells. Cells doubly positive for ERα and AR were abundantly found throughout the MPOA, as reported for rat MPN^37^. In general, the density of doubly ERα- and AR-positive cells was greater than that of singly immunoreactive cells for ERα or AR in the MPOA. Especially, a high proportion of doubly ERα- and AR-positive cells was found in the cMPOA, MPNma, MPNp and MPNc, where neurons intensely expressed c-Fos during the parental, sexual and aggressive behaviors^10^.

Since *Vglut1* and *Vglut3* expressions are low in the preoptic area, glutamatergic neurons are identified by the expression of *Vglut2* mRNA^38, 39^. Consistent with a general dichotomy between excitatory glutamatergic neurons and inhibitory GABAergic neurons, we previously showed that *Gad67*-expressing cells were distinct from *Vglut2*-expressing cells in the MPOA^10^. In most MPOA subregions, the density of *Gad67*-expressing neurons was higher than *Vglut2*-expressing excitatory neurons by at least two-fold, consistent with our previous study in female mice^18^.

In general, neurons that produce neuropeptides co-release fast neurotransmitters such as glutamate or GABA. For example, 90% of galaninergic neurons in the MPOA are *Gad67*-positive[24]. Thus, GABAergic neurons in the MPOA may be classified into *Gad67*(+)-, galanin(+)-neurons and *Gad67*(+)-, galanin(-)-neurons. Given that the combined density of *Gad67*-expressing cells and *Vglut2*-expressing cells is the total density of neurons, there should be a group of neurons that express multiple neuropeptides because the total density of neurons that express neuropeptides surpasses the density of cells that express *Gad67* or *Vglut2* in the cMPOA, MPNvl and BNSTv/mg. The expression of multiple neuropeptides has been reported for orexin neurons that express dynorphin and amylin^40^.

The present study showed that the ratio of ERα- and AR-immunoreactivity largely varied depending on neuronal groups in a subregion-specific manner. Among the neuropeptides examined, *neurotensin*-positive cells showed the highest proportion of ERα- and AR-immunoreactivities throughout the MPOA. Seventy to ninety percent of *neurotensin*-expressing cells were positive for ERα and AR in the cMPOA, MPNvl, MPNc and BNSTv/mg, which indicates that a large proportion of *neurotensin*-expressing cells expressed both ERα and AR in these subregions. Estradiol treatment induced *neurotensin* expression in the MPOA of ovariectomized female rats^41^, suggesting that ERα is required for the proper expression of *neurotensin*. Importantly, *neurotensin* mRNA expression in the rat MPN exhibits a male dominant, sexually dimorphic pattern^23^. Although the detection methods were not consistent, a larger cluster of *neurotensin*-expressing cells was found in the male MPNvl, whereas the cluster of *neurotensin*-expressing cells in the MPNvl of female mice was relatively small^18^.

*Neurotensin*-expressing cells positive for both ERα and AR were abundant in the cMPOA and MPNvl, which exhibited c-Fos expression during male sexual behavior and paternal behavior^10^. Thus, *neurotensin*-expressing cells in the cMPOA and MPNvl may modulate ERα- and AR-dependent male behaviors. Consistently, most *neurotensin*-expressing neurons in the MPOA send their fibers to the ventral tegmental area^42, 43^, and stimulation of *neurotensin*-expressing neurons increased dopamine release in the nucleus accumbens^42^. Since the ventral tegmental area-nucleus accumbens system is involved in male sexual behavior^44^, the current finding of high ERα/AR-positivity of *neurotensin*-expressing cells suggests that *neurotensin*-expressing cells incorporate a hormonal milieu to form sexual/social responses. It was recently reported that *neurotensin*-expressing cells in the MPOA of female mice exhibited an intracellular calcium increase in response to male odor, a social cue with reproductive relevance^42^. Importantly, estradiol treatment enhanced the response of *neurotensin*-expressing cells to male urine odor^42^ suggesting that *neurotensin*-expressing cells in the MPOA work as a regulatory hub for social behaviors of both male and female mice.

Similar to *neurotensin*, 70–90% of *galanin*-expressing cells were positive for ERα and AR in the MPNma, MPNp and MPNc, suggesting a proportion of *galanin*-expressing cells doubly positive for ERα and AR in these subregions. It is also known that *galanin* gene expression is regulated by an estrogen responsive element^45, 46^. *Tac2*-positive cells also showed very high immunoreactivity for gonadal steroid receptors. For example, approximately 70% of the *Tac2*-positive cells in the ACN, cMPOA and MPNvl were immunoreactive for ERα and AR, which indicates at least half of the cells were double positive for ERα and AR. *Penk*-positive cells also showed very high immunoreactivity for gonadal steroid receptors in many MPOA subregions. Thus, androgen may be deeply involved in the MPOA function mediated by cells that express *neurotensin*, *galanin, Tac2* and *Penk*. Collectively, double-labeled cells of gonadal steroid receptors and neuropeptides were abundantly found in the cMPOA, MPNvl and MPNma, which are involved in sexual behavior, paternal behavior and aggressive behaviors, at least in terms of c-Fos expression^10^. Further studies are necessary to examine how mouse behaviors are regulated by gonadal steroid receptor-expressing cell clusters found in specific subregions such as *Cart*-expressing cell clusters in the BNSTpr and MPNmp. The vast majority of *Trh*-expressing cells were negative for ERα and AR, and *Trh*-expressing cells are absent in the cMPOA and MPNvl, which are associated with gonadal steroid-related sexual and paternal behaviors^10^. These findings suggest that the MPOA is incorporated in two neuroendocrine axes for gonadal and thyroid hormones.

In the present study, we quantitated the density of positive cells in brain sections for which single immunostaining or simultaneous staining for protein and mRNA was performed. The sum of the cells doubly positive for *Gad67* and ERα and the cells doubly positive for *Vglut2* and ERα was similar to the total density of ERα-positive cells identified via double immunostaining of ERα and AR. Similarly, the total number of AR positive cells was consistent between single immunostaining and double immunostaining with *in situ* hybridization. In addition, the density profiles of ERα and AR among the MPOA subregions were similar between *in situ* hybridization (ISH) sections and IHC sections (Figs 2, 3, 4, 6, 7). In the preliminary study, we confirmed the expression pattern was similar even when the concentrations of antibodies or riboprobes were changed. These results indicate that our histological procedure and cell counting method are very robust despite technical differences.

In addition to sexual behavior and aggression, the MPOA has multiple roles such as thermoregulation^47, 48^, sleep^49, 50^, body weight regulation^51^ and feeding^52, 53^. Although most lesion and/or pharmacological studies have targeted the entire MPOA in mice, our report on the role of the cMPOA in paternal behavior supports the idea that each MPOA subregion has a distinct functional role^18^. Furthermore, galanin neurons of the MPOA have crucial roles in paternal behavior^24^, and neurotensin neurons of the female MPOA reacted to male odor to activate the reward circuits^42^. These studies identified each neural subtype as an additional dimension of MPOA functional organization. Therefore, both subregions and neuron subtypes are necessary to fully elucidate the functional organization of the MPOA. In fact, GABAergic neurons in the cMPOA subregion are associated with the action of paternal caring versus infanticide^10^.

The present study showed a high degree of co-expression of ERα/AR and neurotransmitters/neuropeptides in the MPOA neurons. This finding implies that gonadal hormones may modulate various functions in which the MPOA is involved, in addition to sexual and paternal behaviors. A recent report indicated that hypothalamic *Pdyn*-expressing neurons expressed amylin, which works synergistically with leptin to inhibit feeding behavior in a sex-dependent manner^40^. Since amylin expression in the MPOA was high in postpartum dams and undetectable in males^54^, gonadal hormones may affect amylin expression in the MPOA, resulting in the modulation of food intake and energy metabolism.

One limitation of the present study is that we only examined young adult male C57BL/6 mice. The expressions of gonadal hormone receptors may differ depending on age, mouse strain and sex. Further studies should incorporate female and aged male mice to determine whether the hormonal milieu and aging may alter gene expression in the MPOA. Another limitation is that the differential expressions of mRNAs and proteins detected by ISH and immunostaining do not always reflect biological differences. To elucidate the functional role of MPOA neuron subtypes, it is essential to use an appropriate Cre-driver mouse for optogenetic and pharmacogenetic analyses. Neuropeptide-specific Cre driver mice are also important to determine how gonadal steroid hormones modulate MPOA neuron subtypes and subsequently alter male behaviors as reported in females^42^. The present study will provide useful information for future studies on the functional anatomy of the MPOA at the subregion level.

## Methods

### Animals and tissue preparation

All procedures were conducted in accordance with the Guidelines for Animal Experiments of Toho University and were approved by the Institutional Animal Care and Use Committee of Toho University (Approved protocol ID #15-52-254). Breeding pairs of C57BL/6 J mice were obtained from Japan SLC Inc. and CLEA Japan. Mice were raised in our breeding colony under controlled conditions (12 h light/dark cycle; lights on at 8:00 A.M.; 23 ± 2 °C; 55 ± 5% humidity; and *ad libitum* access to water and food). Mice were weaned at 4 weeks of age and were housed in groups of four or five.

Male mice (10–20-week-old, n = 39) were anesthetized with sodium pentobarbital (50 mg/kg, i.p.), and then transcardially perfused with 4% paraformaldehyde (PFA) in phosphate buffered saline (PBS). The brains were postfixed in 4% PFA at 4 °C overnight, followed by cryoprotection in 30% sucrose in PBS for two days, embedded in Surgipath (FSC22, Leica Biosystems), and stored at −80 °C until use. The brains were cryosectioned coronally at a thickness of 40 μm.

### Single or double immunohistochemistry for ERα and AR

For single immunostaining for ERα or AR, brain sections were washed and incubated with rabbit anti-ERα (1:5000, C1355, Millipore) or anti-AR (1:500, sc-816, Santa Cruz biotechnology) antibodies, of which the specificities were verified using gene-deficient mice, shRNA-based gene knockdown or preabsorption with antigen^12, 13, 55, 56^. The sections were washed and immersed in Alexa568-conjugated donkey anti-rabbit IgG antibody (1:250, A10037, Thermo scientific) and Hoechst 33342 (1 μg/ml).

To examine the coexpression of ERα and AR, we performed double fluorescent immunostaining using two rabbit antibodies for ERα and AR according to a previously published protocol with modifications for multiple labeling with antibodies that were raised in the same host species^57–59^. In our protocol, a dinitrophenyl-conjugated rabbit anti-AR antibody was used after forming a complex of rabbit anti-ERα antibody and anti-rabbit IgG antibody, which prevents anti-rabbit IgG antibody from binding to rabbit anti-AR antibody.

The anti-AR antibody (20 μg/100 μl) was labeled with dinitrophenyl via incubation with 2.6 μg n-succinimidyl 6-(2,4-dinitroanilino) hexanoate in PBS for 2 hours and purified by ultrafiltration using an Amicon Ultra-0.5 (UFC5050, Merck Millipore). The brain sections were washed with PBS that contained 0.2% Triton X-100 (PBST), incubated with methanol for 5 minutes, and washed with PBST. The sections were blocked with 0.8% Block Ace (Dainihon-Seiyaku, Japan) for 30 minutes in PBST, and then incubated at 4 °C overnight in anti-ERα antibody (1:3000) diluted in 0.4% Block Ace/PBST. The sections were washed and incubated in Fab fragment of Alexa488-conjugated donkey anti-rabbit IgG antibody (1:1000, 711-547-003, Jackson Immunoresearch) for 1 hour. The sections were washed and blocked with 0.8% Block Ace for 30 min in PBST, and incubated at 4 °C overnight in the dinitrophenyl-conjugated anti-AR antibody (1:100). The sections were washed and incubated in a goat anti-dinitrophenyl antibody (1:1000, D9781, Sigma Aldrich) for 1 hour, followed by washing and incubation in a cocktail of Alexa568-conjugated donkey anti-goat IgG antibody (1:1000, ab175704, Abcam) and Hoechst 33342 (1 μg/ml). The sections were mounted on a glass slide with Gel/Mount (BioMeda).

### *In situ* hybridization (ISH) combined with immunohistochemistry

To assess the expression of ERα or AR in each neuronal subtype in the MPOA subregions, we performed two different types of histological examination using serial sections: 1) immunostaining for ERα or AR combined with double ISH for two from ten neuronal subtype markers such as *Gad67*, *Vglut2*, *Pdyn*, *Penk*, *Cart*, *neurotensin*, *galanin*, *Tac1*, *Tac2* and *Trh*, and 2) double ISH combined with immunostaining for markers that are differentially expressed among the MPOA subregions such as calbindin, *neurotensin*, oxytocin, *Penk* and *Vglut2*.

The complete list of riboprobes used for ISH is available in Supplementary Table S1. All cDNA fragments were amplified, inserted into the pGEM-T plasmid (A3600, Promega) and transformed to DH5α *E. coli*. The template cDNA was produced using polymerase chain reaction with the specific primers (5′-ATTTAGGTGACACTATAG-3′) and (5′-TAATACGACTCACTATAGGG-3′). The antisense probes were transcribed by SP6 RNA polymerase (P1085, Promega) in the presence of digoxigenin-labeled UTP (Dig labeling mix; Roche Diagnostics,) or fluorescein-labeled UTP (Fluorescein labeling mix; Roche Diagnostics). The sense probes for control staining were transcribed by T7 RNA polymerase (10881767001, Roche Diagnostics) in the same manner.

For immunostaining for ERα or AR combined with double ISH for neuronal subtype markers, the brain sections were processed for ISH as previously described^10, 18^ with modifications. Briefly, the sections were washed with PBS containing 0.1% Tween-20 (PBT) and postfixed with 4% PFA in PBS for 10 minutes. The sections were immersed in methanol containing 0.3% H_2_O_2_ for 10 minutes, followed by acetylation with 0.25% acetic anhydride in 0.1 M triethanolamine (pH 8.0). The hybridization solution contained 50% deionized formamide, 5 × standard saline citrate (SSC, pH 7.0), 5 mM ethylene-diaminetetraacetic acid (pH 8.0), 0.2 mg/ml yeast tRNA, 0.2% Tween-20, 0.2% sodium dodecyl sulfate, 10% dextran sulfate and 0.1 mg/ml heparin. The sections were prehybridized at 58 °C in the mixture of the hybridization solution and PBT (1:1) for 30 minutes, immersed in the hybridization solution for 15 minutes, and then hybridized with the digoxigenin-labeled and fluorescein-labeled riboprobes (1 μg/ml) at 58 °C for 16 hours. After hybridization, the sections were washed twice with 2 × SSC containing 50% formamide at 58 °C for 10 minutes, incubated with RNAse A solution (20 μg/ml, Sigma) and avidin (0.1 μg/ml) at 37 °C for 60 minutes, rinsed twice in 2 × SSC and rinsed four times in 0.2 × SSC at 37 °C (10 minutes each). The sections were incubated in a peroxidase-conjugated anti-digoxigenin antiserum (1:1000, Roche Diagnostics) with biotin (0.5 μg/ml). After two hours of incubation in the antibody solution at room temperature, the sections were washed and immersed in 0.1 M boric buffer (pH 8.5) containing 4 μM biotin-labeled tyramide, 4% dextran sulfate, 0.05 mg/ml iodophenol, and 0.003% H_2_O_2_ for 30 minutes, followed by incubation with 10% H_2_O_2_/methanol for 30 minutes to quench the peroxidase activity.

The sections were subsequently washed and incubated in a cocktail of anti-ERα (1:5000) or anti-AR (1:500) antibody and peroxidase-conjugated anti-fluorescein antibody (1:1000, Roche Diagnostics) at 4 °C overnight. The sections were washed and immersed in 0.1 M boric buffer (pH8.5) containing 10 μM Alexa488-labeled tyramide, 10% dextran sulfate, 0.05 mg/ml iodophenol and 0.003% H_2_O_2_ for 30 minutes. They were subsequently immersed in a cocktail of Alexa647-conjugated streptavidin (1:10000, Life Technologies), Alexa568-conjugated donkey anti-rabbit IgG antibody (1:250, A10037, Thermo scientific) and Hoechst 33342 (1 μg/ml). The sections were mounted on a glass slide with Gel/Mount.

To identify the MPOA subregions, we performed Nissl staining and double ISH combined with immunostaining for regional markers as previously described. The combinations of reginal marker were 1) double ISH for *neurotensin* and *Penk* combined with immunostaining for calbindin (1:1000, C9848, Sigma-Aldrich), and 2) ISH for *Vglut2* combined with immunostaining for oxytocin (1:5000, #20068, ImmunoStar).

### Histological analysis

Detailed histological analyses of the MPOA for ERα and AR were performed using a set of three sections 120 μm apart between bregma + 0.10 mm and bregma −0.14 mm (corresponds to Fig. 1a-c), where abundant AR- and ERα-positive cells are recognized. Each set of three sections was used for double immunostaining for ERα and AR, or ISH combined with immunostaining, and then processed for positive cell counting. To evaluate the cell density of ERα- or AR-positive cells, three or four sets of MPOA sections from different mice were used for ERα- and AR-immunostaining, and then ERα- and AR-positive cells were counted and averaged. The areas quantitated are presented in Fig. 1a-c as shaded areas.

Fluorescent photographs were obtained using a Nikon Eclipse Ni microscope equipped with the A1R confocal detection system under a 20 × objective (Nikon Instruments Inc., Tokyo, Japan). Each image was obtained as a five-layer z-stack of images, and the optical thickness of the sections was 1.0 μm. Experimental controls were prepared in which one or both primary antibodies were omitted from the reaction solution to confirm no detectable signal. In addition, specific staining of each antisense probe was not observed in the sections stained with the sense probes. For the immunostained sections, some non-specific granule-like signals were identified, however, they were easily distinguished from nuclear or cytoplasmic specific staining.

Images were analyzed using ImageJ software (version 1.50i, NIH, USA). The threshold was determined to be above background or nonspecific signals on the control sections, and the same threshold was used through the analysis for all samples. All procedures for brain sampling, ISH and immunohistochemistry were performed in exactly the same time course under a controlled temperature, thus, the fixed threshold worked to evaluate the positive cells of different mice. Singly or doubly positive cells were manually marked on the threshold images and automatically counted. The same contours for the MPOA subregions as shown in Fig. 1a–c were used throughout all samples with positional adjustments along the dorsal-ventral axis between the anterior commissure and optic tract. All histological procedures were conducted under blind conditions. The data in each subregion were presented as the mean ± S.E.M.

### Data availability

The datasets generated during and/or analysed during the current study are available from the corresponding author on reasonable request.

## Electronic supplementary material

Supplementary information
