# Supplementary material for: Neurotransmitters and neuropeptides in gonadal steroid receptor-expressing cells in medial preoptic area subregions of the male mouse
Source: Sci Rep. 2017 Aug 29;7:9809. doi: 10.1038/s41598-017-10213-4 (PMC5575033; doi:10.1038/s41598-017-10213-4)
Supplement: Supplementary file 1 — Supplementary information [file 41598_2017_10213_MOESM1_ESM.pdf]

Supplementary information

Title of the manuscript:

Neurotransmitters and neuropeptides in gonadal steroid receptor-expressing cells in medial preoptic area subregions of the male mouse

Authors:

Yousuke Tsuneoka, Sachine Yoshida, Kenkichi Takase, Satoko Oda, Masaru Kuroda, Hiromasa Funato

Table. S1 List of riboprobes used in this study

Gene symbol	Gene name	Genbank ID	Range (bp)
Gad67	glutamate decarboxylase 1	NM_008077.4	396-3219
Vglut2	solute carrier family 17, member 6	NM_080853.3	2424-4101
Pdyn	prodynorphin	NM_018863.4	121-2107
Penk	preproenkephalin	NM_001002927.2	114-1407
Cart	CART prepropeptide	NM_013732.7	37-823
Neurotensin	neurotensin	NM_024435.2	11-973
Galanin	galanin	NM_010253.3	88-694
Tac1	tachykinin 1	NM_009311.2	17-935
Tac2	tachykinin 2	NM_001199971.1	156-777
Trh	thyrotropin releasing hormone	NM_009426.3	88-990

Table. S2 Regional density and proportion of double-labeled cells of ERα and AR in each subregion. Number of double-labeled cells among single-labeled cells and its proportion.

	ERα + AR+ / ERα +	ERα + AR+ / ARα
ACN	495 ± 80 / 720 ± 59<br>(68 ± 6 %)	495 ± 80 / 1261 ± 172<br>(39.7 ± 5.7 %)
cMPOA	908 ± 158 / 1270 ± 202<br>(71.1 ± 3.6 %)	908 ± 158 / 1537 ± 259<br>(59 ± 2.7 %)
dmMPOA	161 ± 9 / 511 ± 87<br>(32.7 ± 3.4 %)	161 ± 9 / 533 ± 97<br>(32.2 ± 5.8 %)
MPNma	1273 ± 193 / 2552 ± 268<br>(49.4 ± 2.2 %)	1273 ± 193 / 1748 ± 341<br>(74 ± 3 %)
MPNmp	628 ± 132 / 1179 ± 191<br>(52.4 ± 2.8 %)	628 ± 132 / 1360 ± 153<br>(45.9 ± 7.5 %)
MPNvl	1002 ± 42 / 1821 ± 85<br>(55.5 ± 5.2 %)	1002 ± 42 / 1689 ± 287<br>(61.7 ± 7 %)
MPNp	1300 ± 240 / 1898 ± 361<br>(68.6 ± 2.2 %)	1300 ± 240 / 2064 ± 371<br>(62.9 ± 5.1 %)
MPNc	1039 ± 92 / 1745 ± 146<br>(60.5 ± 8.4 %)	1039 ± 92 / 1893 ± 171<br>(55.3 ± 5 %)
vMPOA	455 ± 108 / 1166 ± 269<br>(39.5 ± 3.1 %)	455 ± 108 / 740 ± 226<br>(67.7 ± 11.9 %)
vlMPOA	68 ± 19 / 244 ± 60<br>(29.9 ± 7.2 %)	68 ± 19 / 415 ± 58<br>(17.3 ± 6 %)
BNSTdm	315 ± 95 / 506 ± 54<br>(59.7 ± 14.3 %)	315 ± 95 / 902 ± 187<br>(33.7 ± 7 %)
BNSTpr	730 ± 124 / 997 ± 153<br>(73.5 ± 5.6 %)	730 ± 124 / 2364 ± 154<br>(30.5 ± 3.3 %)
BNSTv/dm	762 ± 108 / 967 ± 164<br>(79.5 ± 2.3 %)	762 ± 108 / 1635 ± 184<br>(46.8 ± 4.2 %)

Table. S3 Regional density and proportion of double-labeled cells with ERα and neurotransmitter/neuropeptide in each subregion. Density (number/mm^2) of double-labeled cells among single-labeled cells and its proportion.

	Cell count	ACN	cMPOA	dmMPOA	MPNma
Gad67	*Gad67*+ ERα+/ *Gad67*+	412 ± 50 / 1363 ± 288 (31 ± 2.8 %)	583 ± 6 / 1196 ± 221 (50 ± 8.8 %)	337 ± 64 / 1324 ± 171 (26 ± 5.7 %)	1397 ± 142 / 1895 ± 230 (74 ± 1.5 %)
	Gad67+ ERα+/ ERα+	412 ± 50 / 602 ± 91 (69 ± 2.1 %)	583 ± 6 / 895 ± 37 (65 ± 3.4 %)	337 ± 64 / 500 ± 41 (67 ± 7.4 %)	1397 ± 142 / 2519 ± 254 (55 ± 0.0 %)
Vglut2	*Vglut2*+ ERα+/ *Vglut2*+	112 ± 9 / 560 ± 83 (21 ± 1.8 %)	321 ± 30 / 968 ± 172 (35 ± 7.3 %)	84 ± 29 / 457 ± 56 (18 ± 6.1 %)	1156 ± 61 / 1698 ± 186 (70 ± 6.3 %)
	Vglut2+ ERα+/ ERα+	112 ± 9 / 746 ± 147 (17 ± 4.6 %)	321 ± 30 / 1329 ± 49 (24 ± 2.9 %)	84 ± 29 / 464 ± 40 (18 ± 5.2 %)	1156 ± 61 / 2369 ± 116 (49 ± 5.0 %)
Pdyn	*Pdyn*+ ERα+/ *Pdyn*+	41 ± 23 / 92 ± 63 (n.a.)	90 ± 30 / 226 ± 71 (39 ± 3.8 %)	24 ± 19 / 75 ± 39 (22 ± 12 %)	7 ± 7 / 92 ± 18 (8.3 ± 8.3 %)
	Pdyn+ ERα+/ ERα+	41 ± 23 / 699 ± 166 (5.3 ± 3.3 %)	90 ± 30 / 1126 ± 119 (7.6 ± 1.9 %)	24 ± 19 / 383 ± 92 (5.1 ± 4.1 %)	7 ± 7 / 1441 ± 157 (0.6 ± 0.6 %)
Penk	*Penk*+ ERα+/ *Penk*+	66 ± 21 / 175 ± 55 (40 ± 15 %)	416 ± 34 / 550 ± 67 (76 ± 4.8 %)	53 ± 10 / 297 ± 62 (20 ± 6.9 %)	657 ± 183 / 716 ± 221 (88 ± 8.7 %)
	Penk+ ERα+/ ERα+	66 ± 21 / 501 ± 67 (14 ± 5.9 %)	416 ± 34 / 1104 ± 104 (38 ± 3.3 %)	53 ± 10 / 386 ± 95 (16 ± 4.9 %)	657 ± 183 / 2226 ± 519 (29 ± 1.9 %)
Cart	*Cart*+ ERα+/ *Cart*+	79 ± 34 / 200 ± 71 (39 ± 9.6 %)	45 ± 18 / 127 ± 36 (34 ± 9.0 %)	76 ± 42 / 233 ± 76 (29 ± 9.4 %)	244 ± 87 / 305 ± 96 (74 ± 8.8 %)
	Cart+ ERα+/ ERα+	79 ± 34 / 789 ± 114 (10 ± 3.4 %)	45 ± 18 / 1162 ± 116 (3.9 ± 1.5 %)	76 ± 42 / 363 ± 36 (19 ± 8.8 %)	244 ± 87 / 2265 ± 288 (12 ± 5.4 %)
Neurotensin (Nts)	*Nts*+ ERα+/ *Nts*+	223 ± 39 / 290 ± 68 (77 ± 8.3 %)	578 ± 56 / 709 ± 94 (82 ± 2.8 %)	55 ± 18 / 147 ± 58 (39 ± 12 %)	206 ± 11 / 360 ± 87 (63 ± 13 %)
	Nts+ ERα+/ ERα+	223 ± 39 / 751 ± 61 (30 ± 6.1 %)	578 ± 56 / 1231 ± 89 (47 ± 2.8 %)	55 ± 18 / 477 ± 15 (12 ± 3.8 %)	206 ± 11 / 2337 ± 624 (10 ± 2.5 %)
Galanin (Gal)	*Gal*+ ERα+/ *Gal*+	252 ± 76 / 399 ± 109 (62 ± 6.0 %)	305 ± 60 / 507 ± 112 (60 ± 8.3 %)	40 ± 8 / 171 ± 26 (23 ± 1.6 %)	955 ± 68 / 1110 ± 142 (86 ± 4.1 %)
	Gal+ ERα+/ ERα+	252 ± 76 / 399 ± 53 (62 ± 16 %)	305 ± 60 / 577 ± 131 (56 ± 9.3 %)	40 ± 8 / 333 ± 65 (12 ± 0.8 %)	955 ± 68 / 2519 ± 79 (39 ± 12 %)
Tac1	*Tac1*+ ERα+/ *Tac1*+	19 ± 4 / 89 ± 29 (24 ± 8.2 %)	39 ± 18 / 97 ± 29 (36 ± 8.3 %)	23 ± 12 / 50 ± 33 (46 ± 29 %)	60 ± 3 / 88 ± 21 (75 ± 14 %)
	Tac1+ ERα+/ ERα+	19 ± 4 / 487 ± 177 (4.8 ± 1.4 %)	39 ± 18 / 748 ± 304 (15 ± 12 %)	23 ± 12 / 348 ± 100 (5.3 ± 2.7 %)	60 ± 3 / 2345 ± 373 (2.7 ± 0.4 %)
Tac2	*Tac2*+ ERα+/ *Tac2*+	334 ± 34 / 437 ± 38 (76 ± 1.4 %)	393 ± 59 / 579 ± 89 (68 ± 3.5 %)	10 ± 10 / 10 ± 10 (n.a.)	11 ± 11 / 49 ± 20 (17 ± 17 %)
	Tac2+ ERα+/ ERα+	334 ± 34 / 599 ± 66 (57 ± 6.2 %)	393 ± 59 / 1151 ± 20 (34 ± 4.9 %)	10 ± 10 / 395 ± 51 (2.2 ± 2.2 %)	11 ± 11 / 3146 ± 178 (0.3 ± 0.3 %)
Trh	*Trh*+ ERα+/ *Trh*+	18 ± 5 / 112 ± 22 (17 ± 4.5 %)	9 ± 5 / 75 ± 25 (10 ± 5.8 %)	10 ± 10 / 111 ± 24 (8.3 ± 8.3 %)	71 ± 60 / 109 ± 86 (n.a.)
	Trh+ ERα+/ ERα+	18 ± 5 / 551 ± 126 (4.2 ± 2.2 %)	9 ± 5 / 808 ± 25 (1.1 ± 0.6 %)	10 ± 10 / 354 ± 94 (1.9 ± 1.9 %)	71 ± 60 / 2212 ± 112 (3.0 ± 2.5 %)

(n.a.): The proportion was not calculated because one sample did not contain any cells positive for a certain neuropeptide in the MPOA subregion.

Table S3 (continued)

Cell count	MPNmp	MPNvl	MPNp	MPNc
Gad67+ ERα+/ *Gad67*+	573 ± 41 / 1249 ± 63 (46 ± 0.9 %)	1104 ± 195 / 1808 ± 271 (61 ± 6.8 %)	1427 ± 192 / 2180 ± 271 (65 ± 5.4 %)	1059 ± 39 / 2168 ± 400 (50 ± 9.2 %)
Gad67+ ERα+/ ERα+	573 ± 41 / 1640 ± 54 (35 ± 3.6 %)	1104 ± 195 / 1669 ± 226 (66 ± 2.8 %)	1427 ± 192 / 1813 ± 150 (78 ± 4.1 %)	1059 ± 39 / 1353 ± 243 (80 ± 12 %)
Vglut2+ ERα+/ *Vglut2*+	528 ± 156 / 1444 ± 423 (38 ± 13 %)	282 ± 115 / 981 ± 133 (27 ± 8.6 %)	243 ± 35 / 544 ± 77 (45 ± 6.7 %)	170 ± 48 / 494 ± 92 (34 ± 8.7 %)
Vglut2+ ERα+/ ERα+	528 ± 156 / 819 ± 37 (64 ± 17 %)	282 ± 115 / 2136 ± 92 (13 ± 5.6 %)	243 ± 35 / 1604 ± 149 (15 ± 1.2 %)	170 ± 48 / 1260 ± 124 (14 ± 4.4 %)
Pdyn+ ERα+/ *Pdyn*+	53 ± 40 / 95 ± 64 (n.a.)	89 ± 50 / 206 ± 69 (35 ± 13 %)	16 ± 11 / 39 ± 22 (50 ± 29 %)	51 ± 26 / 97 ± 32 (40 ± 20 %)
Pdyn+ ERα+/ ERα+	53 ± 40 / 594 ± 76 (9.9 ± 8.1 %)	89 ± 50 / 1824 ± 126 (5.0 ± 3.0 %)	16 ± 11 / 1023 ± 112 (1.5 ± 0.9 %)	51 ± 26 / 770 ± 284 (5.4 ± 2.9 %)
Penk+ ERα+/ *Penk*+	65 ± 38 / 147 ± 68 (39 ± 5.6 %)	420 ± 24 / 495 ± 40 (85 ± 2.2 %)	111 ± 34 / 188 ± 68 (59 ± 18 %)	219 ± 76 / 284 ± 114 (83 ± 8.6 %)
Penk+ ERα+/ ERα+	65 ± 38 / 908 ± 225 (6.4 ± 2.4 %)	420 ± 24 / 2046 ± 183 (21 ± 2.1 %)	111 ± 34 / 1756 ± 431 (6.0 ± 1.1 %)	219 ± 76 / 790 ± 233 (29 ± 5.9 %)
Cart+ ERα+/ *Cart*+	255 ± 64 / 486 ± 97 (50 ± 6.5 %)	75 ± 34 / 164 ± 57 (40 ± 9.3 %)	87 ± 15 / 191 ± 20 (45 ± 3.6 %)	161 ± 86 / 286 ± 109 (47 ± 17 %)
Cart+ ERα+/ ERα+	255 ± 64 / 707 ± 187 (41 ± 12 %)	75 ± 34 / 1890 ± 290 (4.1 ± 2.1 %)	87 ± 15 / 1368 ± 376 (7.3 ± 1.5 %)	161 ± 86 / 1248 ± 141 (13 ± 6.6 %)
Nts+ ERα+/ *Nts*+	103 ± 25 / 153 ± 25 (69 ± 7.3 %)	1185 ± 52 / 1438 ± 161 (84 ± 5.1 %)	495 ± 234 / 589 ± 282 (88 ± 7.2 %)	489 ± 239 / 544 ± 246 (76 ± 18 %)
Nts+ ERα+/ ERα+	103 ± 25 / 1036 ± 246 (10 ± 1.9 %)	1185 ± 52 / 2181 ± 69 (54 ± 0.7 %)	495 ± 234 / 1764 ± 543 (24 ± 6.4 %)	489 ± 239 / 1513 ± 486 (27 ± 10 %)
Gal+ ERα+/ *Gal*+	441 ± 60 / 794 ± 221 (54 ± 17 %)	262 ± 33 / 441 ± 72 (60 ± 6.9 %)	218 ± 50 / 311 ± 79 (71 ± 6.8 %)	317 ± 43 / 434 ± 90 (73 ± 11 %)
Gal+ ERα+/ ERα+	441 ± 60 / 1075 ± 273 (39 ± 7.9 %)	262 ± 33 / 1031 ± 223 (28 ± 6.9 %)	218 ± 50 / 1105 ± 40 (20 ± 4.8 %)	317 ± 43 / 809 ± 146 (42 ± 10 %)
Tac1+ ERα+/ *Tac1*+	7 ± 7 / 38 ± 27 (n.a.)	35 ± 6 / 102 ± 26 (37 ± 10 %)	33 ± 17 / 55 ± 27 (53 ± 29 %)	10 ± 10 / 78 ± 30 (8.3 ± 8.3 %)
Tac1+ ERα+/ ERα+	7 ± 7 / 1216 ± 842 (0.3 ± 0.3 %)	35 ± 6 / 1287 ± 354 (3.4 ± 1.5 %)	33 ± 17 / 1635 ± 629 (1.6 ± 0.9 %)	10 ± 10 / 914 ± 331 (0.7 ± 0.7 %)
Tac2+ ERα+/ *Tac2*+	23 ± 23 / 30 ± 30 (n.a.)	159 ± 50 / 199 ± 59 (78 ± 3.7 %)	234 ± 17 / 304 ± 33 (78 ± 3.0 %)	152 ± 22 / 202 ± 57 (81 ± 11 %)
Tac2+ ERα+/ ERα+	23 ± 23 / 1352 ± 91 (1.8 ± 1.8 %)	159 ± 50 / 2373 ± 136 (6.6 ± 2.0 %)	234 ± 17 / 2531 ± 102 (9.3 ± 0.7 %)	152 ± 22 / 1643 ± 399 (9.9 ± 1.7 %)
Trh+ ERα+/ *Trh*+	19 ± 11 / 167 ± 68 (11 ± 5.8 %)	15 ± 15 / 62 ± 47 (n.a.)	62 ± 37 / 125 ± 62 (45 ± 23 %)	0 ± 0 / 45 ± 13 (n.a.)
Trh+ ERα+/ ERα+	19 ± 11 / 1390 ± 210 (1.7 ± 1.1 %)	15 ± 15 / 1400 ± 196 (1.4 ± 1.4 %)	62 ± 37 / 1450 ± 190 (4.1 ± 2.1 %)	0 ± 0 / 990 ± 80 (0 ± 0 %.)

(n.a.): The proportion was not calculated because one sample did not contain any cells positive for a certain neuropeptide in the MPOA subregion.

Table S3 (continued)

Cell count	vMPOA	vlMPOA	BNSTdm	BNSTpr	BNSTv/mg
Gad67+ ERα+/ *Gad67*+	990 ± 95 / 1838 ± 164 (54 ± 4.4 %)	128 ± 2 / 900 ± 32 (14 ± 0.2 %)	370 ± 23 / 1687 ± 278 (23 ± 4.5 %)	825 ± 306 / 2594 ± 828 (33 ± 15 %)	753 ± 105 / 1458 ± 264 (52 ± 9.1 %)
Gad67+ ERα+/ ERα+	990 ± 95 / 1550 ± 43 (64 ± 4.4 %)	128 ± 2 / 332 ± 4 (38 ± 0.2 %)	370 ± 23 / 376 ± 17 (98 ± 1.7 %)	825 ± 306 / 1118 ± 371 (73 ± 3.2 %)	753 ± 105 / 961 ± 131 (78 ± 0.3 %)
Vglut2+ ERα+/ *Vglut2*+	165 ± 68 / 598 ± 114 (26 ± 7.3 %)	122 ± 38 / 956 ± 94 (12 ± 2.8 %)	9 ± 5 / 105 ± 17 (9.7 ± 5.0 %)	81 ± 20 / 201 ± 71 (47 ± 20 %)	142 ± 27 / 413 ± 101 (36 ± 7.0 %)
Vglut2+ ERα+/ ERα+	165 ± 68 / 1559 ± 205 (9.9 ± 2.9 %)	122 ± 38 / 496 ± 36 (26 ± 8.6 %)	9 ± 5 / 863 ± 45 (1.1 ± 0.5 %)	81 ± 20 / 1154 ± 78 (7.0 ± 1.6 %)	142 ± 27 / 1084 ± 170 (14 ± 3.8 %)
Pdyn+ ERα+/ *Pdyn*+	48 ± 18 / 76 ± 20 (58 ± 8.3 %)	0 ± 0 / 44 ± 24 (0.0 %.)	35 ± 7 / 87 ± 35 (46 ± 9.7 %)	28 ± 3 / 73 ± 30 (47 ± 12 %)	37 ± 12 / 62 ± 16 (56 ± 12 %)
Pdyn+ ERα+/ ERα+	48 ± 18 / 1286 ± 231 (4.2 ± 2 %)	0 ± 0 / 483 ± 65 (0.0 %.)	35 ± 7 / 602 ± 71 (6.0 ± 1.6 %)	28 ± 3 / 1165 ± 227 (2.7 ± 0.7 %)	37 ± 12 / 690 ± 31 (5.6 ± 1.9 %)
Penk+ ERα+/ *Penk*+	46 ± 13 / 87 ± 26 (53 ± 12 %)	50 ± 33 / 124 ± 61 (29 ± 18 %)	37 ± 20 / 157 ± 74 (30 ± 11 %)	119 ± 9 / 161 ± 15 (74 ± 1.9 %)	110 ± 56 / 235 ± 83 (43 ± 15 %)
Penk+ ERα+/ ERα+	46 ± 13 / 1821 ± 396 (2.6 ± 0.4 %)	50 ± 33 / 466 ± 65 (10 ± 5.7 %)	37 ± 20 / 852 ± 122 (4.3 ± 2.0 %)	119 ± 9 / 1425 ± 173 (8.8 ± 1.9 %)	110 ± 56 / 1044 ± 215 (9.3 ± 3.3 %)
Cart+ ERα+/ *Cart*+	32 ± 24 / 55 ± 38 (n.a.)	41 ± 13 / 249 ± 39 (16 ± 3.4 %)	23 ± 13 / 68 ± 29 (28 ± 17 %)	357 ± 55 / 737 ± 121 (49 ± 5.2 %)	85 ± 34 / 155 ± 57 (53 ± 1.9 %)
Cart+ ERα+/ ERα+	32 ± 24 / 1396 ± 265 (1.8 ± 1.1 %)	41 ± 13 / 413 ± 65 (9.5 ± 1.6 %)	23 ± 13 / 760 ± 184 (5.0 ± 3.8 %)	357 ± 55 / 885 ± 128 (42 ± 5.7 %)	85 ± 34 / 748 ± 159 (11 ± 4.7 %)
Nts+ ERα+/ *Nts*+	269 ± 116 / 413 ± 194 (64 ± 13 %)	143 ± 36 / 222 ± 65 (65 ± 9.0 %)	47 ± 14 / 163 ± 26 (28 ± 5.9 %)	20 ± 13 / 31 ± 19 (58 ± 30 %)	465 ± 127 / 577 ± 167 (81 ± 3.4 %)
Nts+ ERα+/ ERα+	269 ± 116 / 1353 ± 230 (18 ± 1.1 %)	143 ± 36 / 478 ± 60 (29 ± 4.0 %)	47 ± 14 / 667 ± 97 (8.1 ± 3.5 %)	20 ± 13 / 1217 ± 75 (1.6 ± 1.0 %)	465 ± 127 / 1194 ± 271 (38 ± 2.7 %)
Gal+ ERα+/ *Gal*+	19 ± 19 / 37 ± 28 (n.a.)	37 ± 9 / 232 ± 63 (17 ± 4.1 %)	46 ± 35 / 69 ± 49 (n.a.)	24 ± 14 / 116 ± 86 (28 ± 20 %)	260 ± 80 / 460 ± 178 (60 ± 6.3 %)
Gal+ ERα+/ ERα+	19 ± 19 / 839 ± 336 (1.3 ± 1.3 %)	37 ± 9 / 230 ± 33 (17 ± 4.9 %)	46 ± 35 / 246 ± 47 (15 ± 11 %)	24 ± 14 / 329 ± 70 (10 ± 7.5 %)	260 ± 80 / 498 ± 79 (49 ± 10 %)
Tac1+ ERα+/ *Tac1*+	9 ± 9 / 34 ± 34 (n.a.)	3 ± 3 / 34 ± 19 (n.a.)	12 ± 7 / 77 ± 44 (19 ± 16 %)	81 ± 36 / 192 ± 62 (38 ± 15 %)	0 ± 0 / 51 ± 32 (n.a.)
Tac1+ ERα+/ ERα+	9 ± 9 / 1410 ± 440 (1.0 ± 1.0 %)	3 ± 3 / 253 ± 42 (1.0 ± 1.0 %)	12 ± 7 / 406 ± 148 (3.9 ± 3.1 %)	81 ± 36 / 686 ± 172 (13 ± 6.8 %)	0 ± 0 / 721 ± 275 (0 ± 0 %)
Tac2+ ERα+/ *Tac2*+	92 ± 34 / 108 ± 50 (92 ± 8.3 %)	86 ± 29 / 458 ± 81 (18 ± 4.1 %)	58 ± 34 / 90 ± 60 (74 ± 22 %)	25 ± 10 / 36 ± 20 (78 ± 22 %)	550 ± 71 / 789 ± 126 (69 ± 7.7 %)
Tac2+ ERα+/ ERα+	92 ± 34 / 1845 ± 453 (4.6 ± 0.9 %)	86 ± 29 / 430 ± 64 (19 ± 3.6 %)	58 ± 34 / 971 ± 126 (7.1 ± 4.9 %)	25 ± 10 / 1321 ± 147 (2.0 ± 0.8 %)	550 ± 71 / 1348 ± 137 (42 ± 7 %)
Trh+ ERα+/ *Trh*+	69 ± 40 / 446 ± 264 (11 ± 5.7 %)	21 ± 21 / 104 ± 42 (12 ± 12 %)	15 ± 15 / 38 ± 29 (n.a.)	21 ± 11 / 81 ± 54 (n.a.)	23 ± 7 / 62 ± 27 (48 ± 26 %)
Trh+ ERα+/ ERα+	69 ± 40 / 1381 ± 102 (5.4 ± 3.4 %)	21 ± 21 / 275 ± 26 (8.3 ± 8.3 %)	15 ± 15 / 381 ± 31 (4.8 ± 4.8 %)	21 ± 11 / 504 ± 165 (5.9 ± 3.6 %)	23 ± 7 / 721 ± 62 (3.3 ± 0.9 %)

(n.a.): The proportion was not calculated because one sample did not contain any cells positive for a certain neuropeptide in the MPOA subregion.

Table.S4 Regional density and proportion of double-labeled cells with AR and neurotransmitter/neuropeptide in each subregion. Density (number/mm^2) of double-labeled cells among single-labeled cells and its proportion.

	Cell count	ACN	cMPOA	dmMPOA	MPNma
Gad67	*Gad67*+ AR+/ *Gad67*+	887 ± 209 / 1678 ± 355 (52 ± 11 %)	1097 ± 195 / 1586 ± 218 (68 ± 4.2 %)	515 ± 27 / 1812 ± 218 (29 ± 3.1 %)	1298 ± 191 / 1801 ± 227 (71 ± 3.2 %)
	Gad67+ AR+/ AR+	887 ± 209 / 1241 ± 328 (73 ± 4.8 %)	1097 ± 195 / 1717 ± 182 (63 ± 5.1 %)	515 ± 27 / 756 ± 138 (72 ± 9.6 %)	1298 ± 191 / 2058 ± 402 (65 ± 4.5 %)
Vglut2	*Vglut2*+ AR+/ *Vglut2*+	232 ± 93 / 536 ± 151 (39 ± 7.6 %)	427 ± 103 / 863 ± 193 (49 ± 3.1 %)	77 ± 34 / 296 ± 98 (24 ± 2.9 %)	739 ± 165 / 1122 ± 214 (65 ± 4.0 %)
	Vglut2+ AR+/ AR+	232 ± 93 / 1527 ± 366 (15 ± 6.7 %)	427 ± 103 / 1588 ± 67 (27 ± 6.8 %)	77 ± 34 / 646 ± 124 (11 ± 3.2 %)	739 ± 165 / 2005 ± 150 (36 ± 6.1 %)
Pdyn	*Pdyn*+ AR+/ *Pdyn*+	30 ± 13 / 76 ± 36 (47 ± 27 %)	242 ± 45 / 308 ± 67 (77 ± 8.7 %)	4 ± 4 / 4 ± 4 (n.a.)	42 ± 21 / 63 ± 31 (56 ± 29 %)
	Pdyn+ AR+/ AR+	30 ± 13 / 1163 ± 188 (2.4 ± 0.67 %)	242 ± 45 / 1801 ± 14 (13 ± 2.5 %)	4 ± 4 / 505 ± 44 (0.9 ± 0.9 %)	42 ± 21 / 2115 ± 99 (1.9 ± 1.0 %)
Penk	*Penk*+ AR+/ *Penk*+	213 ± 41 / 314 ± 61 (67 ± 8.2 %)	374 ± 10 / 447 ± 50 (85 ± 6.9 %)	49 ± 7 / 285 ± 25 (17 ± 2.0 %)	601 ± 186 / 710 ± 188 (82 ± 4.7 %)
	Penk+ AR+/ AR+	213 ± 41 / 1084 ± 189 (20 ± 1.9 %)	374 ± 10 / 1694 ± 138 (22 ± 1.2 %)	49 ± 7 / 516 ± 162 (13 ± 5.5 %)	601 ± 186 / 1736 ± 230 (33 ± 6.8 %)
Cart	*Cart*+ AR+/ *Cart*+	102 ± 25 / 504 ± 135 (20 ± 0.4 %)	95 ± 32 / 206 ± 97 (50 ± 7.6 %)	59 ± 19 / 134 ± 48 (45 ± 18 %)	290 ± 45 / 424 ± 67 (68 ± 0.3 %)
	Cart+ AR+/ AR+	102 ± 25 / 915 ± 440 (16 ± 11 %)	95 ± 32 / 1427 ± 202 (6.4 ± 1.3 %)	59 ± 19 / 596 ± 285 (15 ± 10 %)	290 ± 45 / 2077 ± 134 (14 ± 3.1 %)
Neurotensin (Nts)	*Nts*+ AR+/ *Nts*+	181 ± 70 / 267 ± 112 (69 ± 1.9 %)	397 ± 19 / 512 ± 40 (78 ± 2.2 %)	32 ± 4 / 72 ± 15 (47 ± 6.7 %)	196 ± 44 / 287 ± 108 (78 ± 14 %)
	Nts+ AR+/ AR+	181 ± 70 / 610 ± 37 (29 ± 9.5 %)	397 ± 19 / 1257 ± 118 (32 ± 3.2 %)	32 ± 4 / 395 ± 163 (12 ± 5.6 %)	196 ± 44 / 1214 ± 228 (16 ± 2.2 %)
Galanin (Gal)	*Gal*+ AR+/ *Gal*+	404 ± 170 / 565 ± 237 (68 ± 15 %)	610 ± 71 / 824 ± 117 (74 ± 6.0 %)	150 ± 44 / 343 ± 120 (49 ± 14 %)	978 ± 21 / 1126 ± 57 (87 ± 2.9 %)
	Gal+ AR+/ AR+	404 ± 170 / 1226 ± 654 (37 ± 4.1 %)	610 ± 71 / 1725 ± 427 (38 ± 5.0 %)	150 ± 44 / 773 ± 291 (24 ± 8.4 %)	978 ± 21 / 1815 ± 198 (55 ± 6.0 %)
Tac1	*Tac1*+ AR+/ *Tac1*+	20 ± 12 / 27 ± 19 (n.a.)	23 ± 3 / 37 ± 6 (62 ± 7.3 %)	21 ± 9 / 44 ± 16 (47 ± 12 %)	25 ± 16 / 25 ± 16 (n.a.)
	Tac1+ AR+/ AR+	20 ± 12 / 798 ± 177 (3.4 ± 2.3 %)	23 ± 3 / 1348 ± 91 (1.7 ± 0.16 %)	21 ± 9 / 305 ± 53 (6.7 ± 2.0 %)	25 ± 16 / 1288 ± 312 (1.9 ± 1.4 %)
Tac2	*Tac2*+ AR+/ *Tac2*+	139 ± 44 / 204 ± 62 (68 ± 1.0 %)	240 ± 24 / 301 ± 43 (80 ± 3.4 %)	16 ± 16 / 16 ± 16 (n.a.)	27 ± 27 / 27 ± 27 (n.a.)
	Tac2+ AR+/ AR+	139 ± 44 / 864 ± 132 (17 ± 7.7 %)	240 ± 24 / 1657 ± 22 (15 ± 1.7 %)	16 ± 16 / 603 ± 5 (2.7 ± 2.7 %)	27 ± 27 / 1910 ± 128 (1.3 ± 1.3 %)
Trh	*Trh*+ AR+/ *Trh*+	38 ± 8 / 148 ± 10 (26 ± 3.9 %)	6 ± 3 / 22 ± 7 (25 ± 14 %)	25 ± 20 / 86 ± 47 (19 ± 10 %)	32 ± 16 / 62 ± 21 (44 ± 22 %)
	Trh+ AR+/ AR+	38 ± 8 / 722 ± 88 (5.4 ± 1.1 %)	6 ± 3 / 1245 ± 235 (0.5 ± 0.3 %)	25 ± 20 / 262 ± 74 (11 ± 7.8 %)	32 ± 16 / 1498 ± 163 (2.0 ± 1.0 %)

(n.a.): The proportion was not calculated because one sample did not contain any cells positive for a certain neuropeptide in the MPOA subregion.

Table S4 (continued)

Cell count	MPNmp	MPNvl	MPNp	MPNc
Gad67+ AR+/ *Gad67*+	1128 ± 139 / 1692 ± 244 (67 ± 3.5 %)	1855 ± 225 / 2390 ± 377 (78 ± 2.7 %)	1916 ± 235 / 2383 ± 299 (80 ± 4.1 %)	1765 ± 128 / 2316 ± 234 (77 ± 2.6 %)
Gad67+ AR+/ AR+	1128 ± 139 / 2056 ± 208 (55 ± 2.5 %)	1855 ± 225 / 2635 ± 191 (70 ± 5.8 %)	1916 ± 235 / 2462 ± 162 (78 ± 4.5 %)	1765 ± 128 / 2366 ± 57 (74 ± 4.1 %)
Vglut2+ AR+/ *Vglut2*+	414 ± 165 / 737 ± 271 (52 ± 8.1 %)	386 ± 136 / 785 ± 271 (49 ± 1.6 %)	189 ± 96 / 293 ± 132 (61 ± 9.5 %)	213 ± 160 / 518 ± 273 (34 ± 11 %)
Vglut2+ AR+/ AR+	414 ± 165 / 1771 ± 62 (24 ± 9.7 %)	386 ± 136 / 2209 ± 82 (17 ± 5.7 %)	189 ± 96 / 2147 ± 369 (7.9 ± 2.9 %)	213 ± 160 / 2087 ± 369 (8.9 ± 6.0 %)
Pdyn+ AR+/ *Pdyn*+	46 ± 17 / 65 ± 27 (67 ± 17 %)	171 ± 17 / 176 ± 22 (98 ± 2.2 %)	51 ± 26 / 85 ± 43 (44 ± 22 %)	31 ± 18 / 31 ± 18 (n.a.)
Pdyn+ AR+/ AR+	46 ± 17 / 1835 ± 90 (2.5 ± 0.9 %)	171 ± 17 / 2033 ± 193 (8.4 ± 0.3 %)	51 ± 26 / 1655 ± 293 (2.7 ± 1.3 %)	31 ± 18 / 2889 ± 192 (1.1 ± 0.58 %)
Penk+ AR+/ *Penk*+	233 ± 56 / 274 ± 83 (83 ± 11 %)	222 ± 58 / 300 ± 106 (76 ± 12 %)	267 ± 30 / 309 ± 57 (88 ± 6.7 %)	299 ± 52 / 381 ± 105 (80 ± 11 %)
Penk+ AR+/ AR+	233 ± 56 / 1828 ± 324 (14 ± 4.3 %)	222 ± 58 / 2343 ± 277 (9.2 ± 1.3 %)	267 ± 30 / 2160 ± 22 (12 ± 1.2 %)	299 ± 52 / 2142 ± 141 (14 ± 3.2 %)
Cart+ AR+/ *Cart*+	336 ± 124 / 660 ± 183 (49 ± 5.1 %)	96 ± 33 / 207 ± 64 (46 ± 15 %)	312 ± 134 / 354 ± 144 (87 ± 2.5 %)	318 ± 12 / 458 ± 24 (70 ± 1.1 %)
Cart+ AR+/ AR+	336 ± 124 / 1304 ± 535 (26 ± 1.3 %)	96 ± 33 / 1998 ± 287 (5.2 ± 2.4 %)	312 ± 134 / 2848 ± 105 (11 ± 5.1 %)	318 ± 12 / 2138 ± 403 (15 ± 2.3 %)
Nts+ AR+/ *Nts*+	57 ± 21 / 99 ± 27 (53 ± 12 %)	985 ± 78 / 1219 ± 143 (81 ± 5.4 %)	240 ± 42 / 328 ± 49 (72 ± 4.0 %)	308 ± 162 / 473 ± 208 (55 ± 12 %)
Nts+ AR+/ AR+	57 ± 21 / 1148 ± 261 (5.1 ± 2.0 %)	985 ± 78 / 1808 ± 201 (55 ± 2.6 %)	240 ± 42 / 1504 ± 214 (17 ± 3.9 %)	308 ± 162 / 1766 ± 286 (17 ± 7.8 %)
Gal+ AR+/ *Gal*+	472 ± 212 / 722 ± 265 (62 ± 6.2 %)	567 ± 59 / 742 ± 89 (77 ± 1.4 %)	422 ± 91 / 481 ± 96 (87 ± 3.4 %)	616 ± 76 / 798 ± 119 (78 ± 3.5 %)
Gal+ AR+/ AR+	472 ± 212 / 1421 ± 332 (34 ± 11 %)	567 ± 59 / 2078 ± 239 (28 ± 4.1 %)	422 ± 91 / 2076 ± 468 (20 ± 0.7 %)	616 ± 76 / 2418 ± 467 (28 ± 8.4 %)
Tac1+ AR+/ *Tac1*+	10 ± 10 / 10 ± 10 (n.a.)	28 ± 7 / 31 ± 10 (89 ± 11 %)	21 ± 4 / 29 ± 8 (72 ± 15 %)	55 ± 14 / 77 ± 25 (72 ± 15 %)
Tac1+ AR+/ AR+	10 ± 10 / 1116 ± 144 (0.7 ± 0.7 %)	28 ± 7 / 1887 ± 94 (1.5 ± 0.3 %)	21 ± 4 / 1365 ± 410 (1.6 ± 0.2 %)	55 ± 14 / 1698 ± 203 (3.4 ± 1.0 %)
Tac2+ AR+/ *Tac2*+	26 ± 2 / 39 ± 14 (75 ± 25 %)	177 ± 43 / 230 ± 49 (76 ± 6.6 %)	86 ± 36 / 129 ± 44 (64 ± 14 %)	50 ± 14 / 107 ± 29 (47 ± 13 %)
Tac2+ AR+/ AR+	26 ± 2 / 1728 ± 98 (1.5 ± 0.0 %)	177 ± 43 / 2131 ± 87 (8.2 ± 1.7 %)	86 ± 36 / 1827 ± 355 (4.5 ± 1.1 %)	50 ± 14 / 1714 ± 357 (3.2 ± 1.5 %)
Trh+ AR+/ *Trh*+	88 ± 45 / 191 ± 84 (42 ± 12 %)	7 ± 4 / 29 ± 5 (22 ± 11 %)	34 ± 22 / 144 ± 89 (35 ± 18 %)	17 ± 9 / 17 ± 9 (n.a.)
Trh+ AR+/ AR+	88 ± 45 / 1188 ± 269 (8.5 ± 4.0 %)	7 ± 4 / 1918 ± 351 (0.46 ± 0.2 %)	34 ± 22 / 1854 ± 306 (1.8 ± 1.3 %)	17 ± 9 / 1626 ± 378 (1.4 ± 0.7 %)

(n.a.): The proportion was not calculated because one sample did not contain any cells positive for a certain neuropeptide in the MPOA subregion.

Table S4 (continued)

Cell count	vMPOA	vlMPOA	BNSTdm	BNSTpr	BNSTv/mg
Gad67+ AR+/ *Gad67*+	816 ± 135 / 1516 ± 331 (55 ± 8.5 %)	239 ± 32 / 937 ± 133 (26 ± 1.9 %)	607 ± 244 / 1747 ± 423 (33 ± 12 %)	2092 ± 204 / 2762 ± 296 (76 ± 2.2 %)	787 ± 41 / 1416 ± 179 (57 ± 6.9 %)
Gad67+ AR+/ AR+	816 ± 135 / 1433 ± 149 (56 ± 3.4 %)	239 ± 32 / 659 ± 37 (36 ± 3.7 %)	607 ± 244 / 675 ± 287 (92 ± 2.6 %)	2092 ± 204 / 2497 ± 193 (84 ± 3.3 %)	787 ± 41 / 1089 ± 101 (73 ± 3.4 %)
Vglut2+ AR+/ *Vglut2*+	138 ± 19 / 464 ± 97 (31 ± 4.9 %)	115 ± 21 / 416 ± 34 (27 ± 3.2 %)	60 ± 16 / 228 ± 47 (26 ± 5.6 %)	118 ± 25 / 235 ± 92 (56 ± 8.3 %)	224 ± 110 / 516 ± 157 (39 ± 8.3 %)
Vglut2+ AR+/ AR+	138 ± 19 / 1741 ± 22 (7.9 ± 1.2 %)	115 ± 21 / 881 ± 126 (13 ± 0.6 %)	60 ± 16 / 593 ± 77 (11 ± 3.1 %)	118 ± 25 / 2878 ± 166 (4.2 ± 1.2 %)	224 ± 110 / 1364 ± 235 (15 ± 5.2 %)
Pdyn+ AR+/ *Pdyn*+	11 ± 11 / 21 ± 21 (n.a.)	3 ± 3 / 13 ± 9 (n.a.)	15 ± 10 / 22 ± 13 (58 ± 30 %)	117 ± 46 / 138 ± 51 (76 ± 13 %)	62 ± 37 / 86 ± 51 (57 ± 30 %)
Pdyn+ AR+/ AR+	11 ± 11 / 1282 ± 320 (1.4 ± 1.4 %)	3 ± 3 / 601 ± 110 (0.8 ± 0.8 %)	15 ± 10 / 536 ± 39 (2.8 ± 1.8 %)	117 ± 46 / 1842 ± 40 (6.4 ± 2.5 %)	62 ± 37 / 1146 ± 228 (4.6 ± 2.8 %)
Penk+ AR+/ *Penk*+	55 ± 43 / 158 ± 83 (34 ± 17 %)	4 ± 4 / 32 ± 15 (11 ± 11 %)	65 ± 9 / 188 ± 39 (36 ± 6.9 %)	258 ± 133 / 351 ± 165 (70 ± 5 %)	214 ± 105 / 359 ± 177 (56 ± 12 %)
Penk+ AR+/ AR+	55 ± 43 / 1123 ± 158 (4.7 ± 3.3 %)	4 ± 4 / 496 ± 169 (0.6 ± 0.6 %)	65 ± 9 / 370 ± 91 (19 ± 2.9 %)	258 ± 133 / 2006 ± 383 (14 ± 7.6 %)	214 ± 105 / 1062 ± 110 (20 ± 11 %)
Cart+ AR+/ *Cart*+	89 ± 68 / 124 ± 76 (60 ± 26 %)	94 ± 19 / 225 ± 37 (42 ± 8.3 %)	92 ± 10 / 158 ± 56 (64 ± 16 %)	424 ± 55 / 732 ± 216 (60 ± 16 %)	95 ± 16 / 189 ± 47 (51 ± 13 %)
Cart+ AR+/ AR+	89 ± 68 / 1442 ± 265 (7.3 ± 6.0 %)	94 ± 19 / 552 ± 89 (18 ± 6.4 %)	92 ± 10 / 621 ± 51 (15 ± 2.9 %)	424 ± 55 / 2351 ± 199 (18 ± 3.9 %)	95 ± 16 / 1679 ± 103 (5.7 ± 1.3 %)
Nts+ AR+/ *Nts*+	160 ± 63 / 304 ± 88 (50 ± 6.7 %)	46 ± 20 / 117 ± 29 (37 ± 9.5 %)	39 ± 11 / 58 ± 23 (73 ± 13 %)	36 ± 21 / 55 ± 40 (n.a.)	527 ± 27 / 676 ± 63 (78 ± 4.3 %)
Nts+ AR+/ AR+	160 ± 63 / 878 ± 194 (19 ± 6.1 %)	46 ± 20 / 375 ± 71 (14 ± 7.4 %)	39 ± 11 / 480 ± 84 (8.3 ± 2.0 %)	36 ± 21 / 1555 ± 71 (2.3 ± 1.3 %)	527 ± 27 / 1087 ± 90 (49 ± 6.1 %)
Gal+ AR+/ *Gal*+	104 ± 33 / 127 ± 35 (79 ± 6.5 %)	88 ± 17 / 353 ± 98 (28 ± 8.7 %)	58 ± 25 / 137 ± 73 (47 ± 6.6 %)	278 ± 202 / 310 ± 223 (91 ± 5.3 %)	415 ± 76 / 595 ± 99 (69 ± 2.3 %)
Gal+ AR+/ AR+	104 ± 33 / 1216 ± 46 (8.7 ± 3.0 %)	88 ± 17 / 616 ± 127 (15 ± 3.5 %)	58 ± 25 / 449 ± 158 (22 ± 14 %)	278 ± 202 / 2340 ± 448 (13 ± 9.5 %)	415 ± 76 / 1318 ± 131 (31 ± 2.7 %)
Tac1+ AR+/ *Tac1*+	0 ± 0 / 9 ± 9 (n.a.)	12 ± 7 / 12 ± 7 (n.a.)	24 ± 13 / 53 ± 26 (45 ± 23 %)	155 ± 29 / 179 ± 37 (87 ± 2 %)	11 ± 5 / 37 ± 10 (22 ± 11 %)
Tac1+ AR+/ AR+	0 ± 0 / 901 ± 48 (0%)	12 ± 7 / 296 ± 77 (3.3 ± 1.7 %)	24 ± 13 / 290 ± 98 (6.2 ± 3.1 %)	155 ± 29 / 1452 ± 71 (11 ± 2.6 %)	11 ± 5 / 1004 ± 235 (1.4 ± 0.8 %)
Tac2+ AR+/ *Tac2*+	40 ± 12 / 95 ± 14 (42 ± 8.3 %)	144 ± 9 / 362 ± 40 (40 ± 4.9 %)	79 ± 66 / 104 ± 79 (59 ± 34 %)	65 ± 14 / 122 ± 24 (53 ± 9.8 %)	346 ± 77 / 545 ± 166 (65 ± 5.7 %)
Tac2+ AR+/ AR+	40 ± 12 / 1505 ± 230 (2.9 ± 1.2 %)	144 ± 9 / 857 ± 214 (18 ± 5.5 %)	79 ± 66 / 474 ± 2 (17 ± 14 %)	65 ± 14 / 2399 ± 604 (3 ± 1.4 %)	346 ± 77 / 1170 ± 170 (31 ± 11 %)
Trh+ AR+/ *Trh*+	92 ± 35 / 462 ± 210 (24 ± 4.8 %)	81 ± 26 / 367 ± 159 (25 ± 6.1 %)	14 ± 14 / 33 ± 24 (n.a.)	13 ± 8 / 30 ± 20 (n.a.)	16 ± 11 / 32 ± 13 (39 ± 20 %)
Trh+ AR+/ AR+	92 ± 35 / 723 ± 129 (15 ± 6.9 %)	81 ± 26 / 502 ± 147 (19 ± 7.1 %)	14 ± 14 / 585 ± 88 (3.3 ± 3.3 %)	13 ± 8 / 1532 ± 152 (0.9 ± 0.6 %)	16 ± 11 / 1122 ± 118 (1.3 ± 0.9 %)

(n.a.): The proportion was not calculated because one sample did not contain any cells positive for a certain neuropeptide in the MPOA subregion.
